# Nearest-neighbor parameters for the prediction of RNA duplex stability in diverse *in vitro* and cellular-like crowding conditions

**DOI:** 10.1093/nar/gkad020

**Published:** 2023-01-31

**Authors:** Saptarshi Ghosh, Shuntaro Takahashi, Dipanwita Banerjee, Tatsuya Ohyama, Tamaki Endoh, Hisae Tateishi-Karimata, Naoki Sugimoto

**Affiliations:** Frontier Institute for Biomolecular Engineering Research (FIBER), Konan University, 7-1-20 Minatojima-Minamimachi, Chuo-ku, Kobe 650-0047, Japan; Frontier Institute for Biomolecular Engineering Research (FIBER), Konan University, 7-1-20 Minatojima-Minamimachi, Chuo-ku, Kobe 650-0047, Japan; Frontier Institute for Biomolecular Engineering Research (FIBER), Konan University, 7-1-20 Minatojima-Minamimachi, Chuo-ku, Kobe 650-0047, Japan; Frontier Institute for Biomolecular Engineering Research (FIBER), Konan University, 7-1-20 Minatojima-Minamimachi, Chuo-ku, Kobe 650-0047, Japan; Frontier Institute for Biomolecular Engineering Research (FIBER), Konan University, 7-1-20 Minatojima-Minamimachi, Chuo-ku, Kobe 650-0047, Japan; Frontier Institute for Biomolecular Engineering Research (FIBER), Konan University, 7-1-20 Minatojima-Minamimachi, Chuo-ku, Kobe 650-0047, Japan; Frontier Institute for Biomolecular Engineering Research (FIBER), Konan University, 7-1-20 Minatojima-Minamimachi, Chuo-ku, Kobe 650-0047, Japan; Graduate School of Frontiers of Innovative Research in Science and Technology (FIRST), Konan University, 7-1-20 Minatojima-Minamimachi, Chuo-ku, Kobe 650-0047, Japan

## Abstract

RNA performs various spatiotemporal functions in living cells. As the solution environments significantly affect the stability of RNA duplexes, a stability prediction of the RNA duplexes in diverse crowded conditions is required to understand and modulate gene expression in heterogeneously crowded intracellular conditions. Herein, we determined the nearest-neighbor (NN) parameters for RNA duplex formation when subjected to crowding conditions with an ionic concentration relevant to that found in cells. Determination of the individual contributions of excluded volume effect and water activity to each of the NN parameters in crowded environments enabled prediction of the thermodynamic parameters and their melting temperatures for plenty of tested RNA duplex formation *in vitro* and in cell with significant accuracy. The parameters reported herein will help predicting RNA duplex stability in different crowded environments, which will lead to an improved understanding of the stability-function relationship for RNAs in various cellular organelles with different molecular environments.

## Graphical Abstract

**Graphical Abstract:**
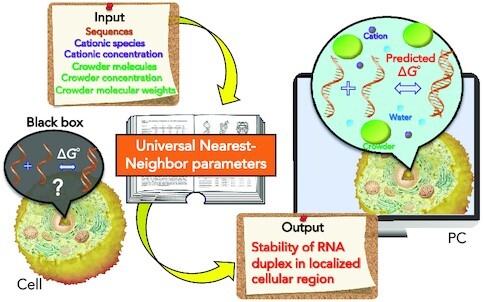
Universal nearest-neighbor parameters have been developed for predicting RNA duplex stability in diverse in vitro crowding conditions and specific cellular regions like cytosol, nucleus and nucleolus.

## INTRODUCTION

RNA is one of the most important biomolecules present in cells and is associated with various biological processes, such as protein synthesis, biocatalysis, and enzymatic reactions (1,2). RNA can form various inter- or intramolecular Watson–Crick base-paired secondary structures sourced from the primary base sequences, and these structures often influence RNA functions. Among the possible RNA secondary structural motifs, the stem-loop is the most common, where the duplex stem is generally formed by Watson–Crick pairing between complementary bases. Such RNA secondary structures have been shown to affect the kinetics and yields of transcription and translation reactions significantly, depending on the stability of these structures (3–5). Furthermore, miRNA, which is a short RNA naturally transcribed in the cells, forms duplexes with mRNA via base pairings and regulates translation efficiency with an RNA-induced silencing complex (RISC) (6). From a biotechnological viewpoint, an siRNA that fully hybridizes with a target mRNA induces the silencing of gene expression (7). Moreover, RNA secondary structures have been widely utilized in the development of diagnosis, therapy, and targeted delivery (8). However, the determination of RNA secondary structures in cells is limited. Certain techniques can be used to probe the base pairings of RNA in cells using chemical foot printing (9), confocal microscopy (10), and in-cell NMR (11), although these techniques are often complicated and time-consuming. Therefore, computational prediction models are ideal and more achievable for studying RNA secondary structure formation in cells.

To predict the formation of an RNA base pair, the thermodynamic stability of Watson–Crick base pair formation is required. The most successful prediction method for duplex stability is based on the nearest-neighbor (NN) model, which assumes that the free energy change (Δ*G*°) for the duplex formation comprises the sum of the Δ*G*° values of adjacent NN base pairs and helix initiation (12,13). Similarly, the relevant thermodynamic parameters, such as changes in enthalpy (Δ*H*°) and entropy (Δ*S*°), can also be predicted from the base sequence. Based on this model, parameters for predicting the thermodynamics of RNA, DNA and RNA/DNA hybrid duplex formations were developed (14–16). Due to the establishment of the NN parameters (14), RNA secondary structure prediction models such as Mfold (17), RNA fold (18), RNA structure (19) and the recently developed MXFold2 (20) and software for RNAs containing modified nucleobase (21) compare the minimum free energies for different possible conformations to obtain the most probable secondary structure. However, these methods have a critical limitation for their application in cells, because the classical NN parameters for RNA duplex formation were determined under conditions of 1 M NaCl (22), whereas the intracellular environment contains much lower and various cation concentrations (23). For example, cytosol consists not only monovalent cations like K^+^ and Na^+^ but also different divalent cations like Mg^2+^ and Ca^2+^(23). Therefore, prediction parameters for RNA duplexes applicable for various cation conditions are required. Furthermore, intracellular environments remain populated with highly concentrated macromolecules termed ‘molecular crowding’ (24). As an example, nucleus contains nucleic acids and proteins such as histones and protamines as the main component of biomolecules, whereas, biomolecular composition in cytosol majorly consists of protein filaments, microtubules, vesicles and polysaccharides (23). Such molecular crowding in cell significantly affects nucleic acid structure and stability (25). To understand the behavior of nucleic acid structures in cells, synthetic cosolutes are often used to mimic intracellular crowding conditions *in vitro* whereby polyethylene glycols (PEGs) are predominantly used (26). Recently, we developed NN parameters for DNA duplexes available in various concentrations of cations and cosolutes (27). For RNA duplexes, however, NN parameters are only available in a specific crowding solution containing 1 M NaCl, limiting their applications for cellular conditions (28). Intracellular RNAs exist in various organelles, such as the cytosol, nucleus, and stress granules. Moreover, intracellular conditions vary dynamically. Therefore, a general method is required to utilize these parameters in varying concentrations of a cosolute, in the presence of different cosolutes, and under physiological cation concentrations.

In this study, we analyzed the thermodynamics of formation of different RNA duplexes with varying lengths and base compositions in a physiologically relevant solution containing 100 mM NaCl and 40 wt% PEG with an average molecular weight of 200 g mol^−1^ (PEG200). Based on these data, we determined the NN parameters of 10 possible propagating Watson–Crick NN base pairs and helix initiation factor for RNA duplexes, which predicted stability of RNA duplexes with high accuracy even in a solution containing exact intracellular cation composition. Furthermore, we expanded these NN parameters available under different cosolutes at varying concentrations by determining the contributions of the excluded volume effect and water activity under the cosolute-containing solutions on duplex stability, which permitted to predict stabilities of different RNA duplexes in diverse *in vitro* and intracellular crowding conditions with significant accuracy. The universal NN parameters applicable to different crowding conditions will be not only useful in RNA structure determination software when attempting to improve our understanding of RNA structures *in vivo*, but also in designing RNA-based therapeutics.

## MATERIALS AND METHODS

### Materials

All the synthetic RNA oligonucleotides used in this work, listed in Table S1 (in Supplementary Data), were purchased from Japan Bio Services Co. and purified by high-performance liquid chromatography (HPLC). Dialysis could be done to set the exact ionic concentrations of the studied solutions, however, we did not perform dialysis, except otherwise mentioned. This is due to the fact that we used quite short length RNAs with low concentrations for this study. Thus, even if there were some residual cations on RNAs after synthesis and purification, the effect on the stability should be negligible. RNA samples were prepared in Milli-Q water and stocked at -20°C until use. The concentrations of the single-stranded oligonucleotides were determined by measuring the absorbance at 260 nm at 90°C using the extinction coefficients (29). Polyethylene glycols (PEGs), glycerol, ethylene glycol, 2-methoxy ethanol, 1,2-dimethoxy ethane and 1,3-propanediol were purchased from Wako Pure Chemical Industries, Japan and used without further purification. Disodium hydrogen phosphate (Na_2_HPO_4_), Dipotassium hydrogen phosphate (K_2_HPO_4_), sodium chloride (NaCl), potassium chloride (KCl), magnesium chloride (MgCl_2_) and calcium chloride (CaCl_2_) were purchased from Wako Pure Chemical Industries (Japan); disodium ethylenediaminetetraacetate (Na_2_EDTA) and dipotassium ethylenediaminetetraacetate (K_2_EDTA) were purchased from Dojindo Molecular Technologies (Japan) and all these chemicals were used as received.

### UV melting measurement

Absorption spectra were measured on a Shimadzu 1800 spectrophotometer with a thermoprogrammer. All the experiments were conducted in a buffer containing 10 mM Na_2_HPO_4_, 1 mM Na_2_EDTA and 100 mM NaCl, in the presence of cosolutes with specific weight percentages. We adjusted the pH of the buffer to 7.0 after adding the cosolutes to maintain the pH of the buffer solution. For melting experiments, 10–12 fresh solutions of oligonucleotides were prepared by varying the concentrations over a 50–150-fold range. The RNA solutions were kept at 90°C for 5 min, followed by the decrease of temperature from 90°C to 0°C at a rate of 1°C min^−1^ to anneal the duplexes. Thereafter, the samples were heated from 0°C to 90°C at a rate of 0.5°C min^−1^ to melt the duplex after keeping them at 0°C for 5 min. Water condensation on the cuvette exterior at low temperature was avoided by flushing with a constant stream of dry N_2_ gas.

### Determination of thermodynamics for duplex formation

Thermodynamic parameters (Δ*H*°, Δ*S*° and Δ*G*°_37_) for RNA duplexes were determined from the *T_m_*^−1^ versus ln (*C*_*t*_/*s*) plots as described in our earlier studies (27,30,31). From the slope and intercept of the linear plots, the thermodynamic parameters were calculated using the following equations:


(1) $$\begin{equation*}{T_m}^{ - 1} = R\ {\rm{ln}}\left( {{C_t}/s} \right)/\Delta H^\circ + \Delta S^\circ /\Delta H^\circ \end{equation*}$$

(2) $$\begin{equation*}\Delta G{^\circ _{37}} = \Delta H^\circ -310.15 \bullet \Delta S^\circ \end{equation*}$$

where *R* is the gas constant, *C*_*t*_ is the total strand concentration of the oligonucleotides, and *s* reflects the sequence symmetry of the self-complementary (*s* = 1) or non-self-complementary strands (*s* = 4). Following the standard practice for calculation of the thermodynamic parameters, we assumed the difference in heat capacities (Δ*C*_p_) of the two states (single-strand and duplex) to be zero (14,22). Because the melting temperature (*T_m_*) values for most of the studied sequences in the crowding condition were not far from 37°C, zero Δ*C*_p_ approximation should be acceptable for Δ*G*°_37_ calculation due to minimal extrapolations. Generally, Δ*G*°_37_ is relatively insensitive to Δ*C*_p_ due to enthalpy–entropy compensation (22,32).

### Calculation of nearest-neighbor parameters

NN parameters were calculated using our original software written in Python based on the linear least square fitting algorithm as described in our earlier studies (27,31). Briefly, the program determined Δ*G*°_37_ and Δ*H*° using a set of 13 parameters (ten Watson-Crick NN base pairs, one initiation parameter, a parameter for terminal AU pair and a symmetry parameter for self-complementary sequences) by applying linear least square fitting to the measured values of the thermodynamic parameters of 45 RNA duplexes in 40 wt% PEG200 with 100 mM NaCl. The parameters for symmetry correction were set to the same value as that in the absence of cosolutes, since it does not depend on the solution condition, but on the complementarity of the sequences. Δ*S*° values were obtained from the determined respective values of Δ*G*°_37_ and Δ*H*° using Equation 2.

### Circular dichroism (CD) measurements

CD spectra were obtained on a JASCO J-1500 spectropolarimeter equipped with a temperature controller. The experimental temperature was 4°C. The cuvette-holding chamber was flushed with a constant stream of dry N_2_ gas to avoid water condensation on the cuvette exterior. The CD spectra were measured from 200 to 340 nm in 1.0 mm path-length cuvettes with a scan rate of 50 nm min^−1^. The concentration of the samples was 20 μM in a buffer containing 100 mM NaCl, 10 mM Na_2_HPO_4_ (pH 7.0) and 1 mM Na_2_EDTA with or without 40 wt% PEG200.

### Water activity and dielectric constant measurement

The water activities were determined by vapor phase osmometry (a Wescor pressure osmometer) or freezing point depression osmometry (a Knauer osmometer). The water activity (*a*_w_) was calculated from the measured osmomolality (mmol kg^−1^) using the following equation (33)


(3) $$\begin{equation*}\Psi = \left( {RT/{M_{\rm w}}} \right){\rm{ ln}}\,{a_{\rm w}}\end{equation*}$$

where Ψ and *M*_w_ represent the water potential and the molecular weight of water (0.018 kg mol^−1^), respectively. The relationship between water potential and osmomolality is given by the following equation (33)


(4) $$\begin{equation*}\Psi \left( {{\rm{MPa}}} \right) = {\rm{osmomolality}}\left( {{\rm{mmol\, k}}{{\rm{g}}^{{\rm{ - 1}}}}} \right) \times {10^3}/\left( { - 400} \right)\end{equation*}$$

The dielectric constants were measured by using fluorescent probe ANS. The fluorescence maximum of ANS showed a hypsochromic shift in the media of low dielectricity. The spectra were recorded in fluorescence spectrophotometer (Jasco F-6500) exciting at 370 nm. The dielectric constants were calculated using a standard curve of organic solvents with known values.

## RESULTS AND DISCUSSION

### Validation of the NN model for RNA duplexes under physiological crowding condition

We selected RNA sequences ranging between 6- and 12-mers, ensuring sufficient stability and two-state transitions, since short sequences are too unstable to analyze, and longer sequences may show non-two-state transitions by forming intramolecular structures. The chosen sequences had different combinations of NN frequencies, covering all 10 NN base pairs for RNA duplex formation, without any bias to particular pairs (Table S1). We selected 25 non-self-complementary (NS1–NS16) and 20 self-complementary (S1–S15) RNA duplexes, as shown in Table S1. Among the duplexes investigated, nine pairs of non-self-complementary sequences (NS7–NS15) and five pairs of self-complementary sequences (S9–S12 and S15) had identical NN base sets (marked as ‘a’ and ‘b’ in Table S1). CD spectral data of representative RNA duplexes indicated that these sequences adopted the A-form duplex both in the absence or presence of PEG200 (Supplementary Figure S1). The slight changes in the peak positions and ellipticities in the PEG200 solution (Supplementary Figure S1) can be attributed to the different stabilities of the RNA duplexes under crowded conditions.

**Figure 1.**
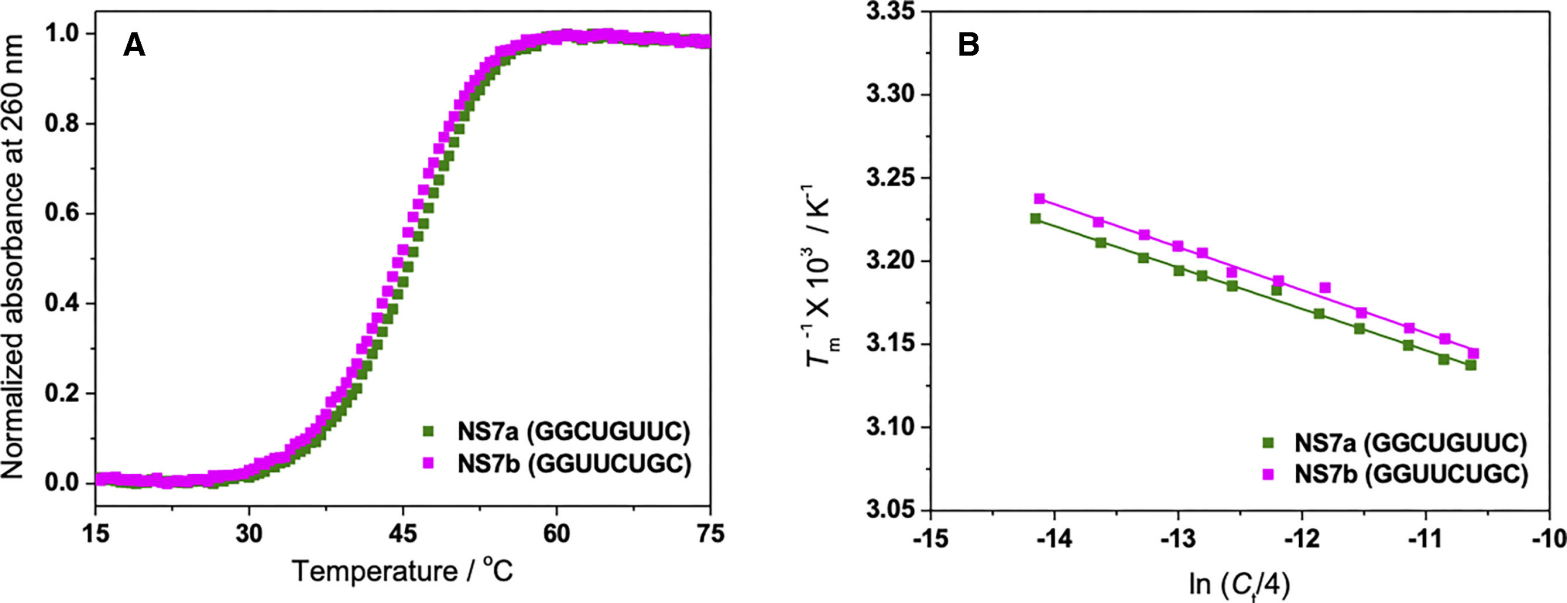
Representative UV melting curves of 100 μM NS7a and NS7b in 40 wt% PEG200 with 100 mM NaCl (**A**) and *T*_m_^−1^ versus ln (*C_t_*/4) plots for these sequences (**B**).

Thermodynamic data (Δ*H*°, Δ*S*° and Δ*G*°_37_) of RNA duplex formation for the studied sequences under physiological crowding condition containing 40 wt% PEG200 with 100 mM NaCl were determined from the UV melting experiment, as depicted in Figure 1, for a pair of typical sequences. Absence of hysteresis between the denaturation and renaturation profiles for all sequences, as shown in Supplementary Figure S2 for representative sequences, indicated that the transition between duplex and single-strands was of a two-state nature. The obtained parameters are presented in Table S2, together with their stabilities in the absence of a cosolute. It should be noted that the actual Na^+^ concentration of the experimental solution was 122 mM considering the Na^+^ derived from the buffer components (10 mM Na_2_HPO_4_ and 1 mM Na_2_EDTA) in addition to 100 mM NaCl. Consistent with previous reports, the stability of all the RNA duplexes decreased under crowding condition due to changes in the solution properties such as water activity and the dielectric constant of the medium in the presence of a cosolute (34,35). The extent of destabilization of RNA duplexes in the cosolute solution varied depending on the base sequences of the duplexes. Moreover, RNA sequences with identical NN sets showed similar melting profiles and *T*_m_^−1^ versus ln (*C*_*t*_/4) plots under physiological crowding condition (Figure 1). As a result, the thermodynamic parameters obtained for the pairs of sequences with the same NN sets were also similar (Table S2). For example, in the case of sequence NS7a (GGCUGUUC) and NS7b (GGUUCUGC), the thermodynamic values were −79.8 and −76.9 kcal mol^–1^ for Δ*H*°, −71.1 and −68.5 kcal mol^–1^ for *T*Δ*S*°, and −8.7 and −8.4 kcal mol^–1^ for Δ*G*°_37_, respectively. For the 14 pairs of RNA sequences with the same NN base sets (sequences indicated by ‘a’ and ‘b’ in Table S2), the average differences in the percentage of measured Δ*H*°, *T*Δ*S*° and Δ*G*°_37_ were 5.2%, 5.8% and 4.0%, respectively. Since the NN model assumes the same stability for sequences with identical NN pairs (36), the results clearly showed the validity of the model for RNA duplexes under physiological crowding condition and the dependency of individual stability of the duplexes on the NN pairs present in that particular sequence.

### NN parameters for RNA duplexes in the physiological crowding condition

The parameters for 10 possible Watson–Crick NN pairs (Δ*H*°_NN_, Δ*S*°_NN_ and Δ*G*°_37, NN_) and the penalties for helix initiation and terminal A–U pairs were calculated using a linear least-square fitting algorithm from the thermodynamic parameters of 45 RNA sequences (Table S2) (27). Table 1 lists the parameters determined in the presence of 40 wt% PEG200 at 100 mM NaCl. The qualitative trend observed in the order of increasing stability (–Δ*G*°_37,NN_) was: r(AU/UA) ≈ r(AA/UU) < r(UA/AU) < r(CU/GA) ≈ r(GU/CA) < r(CA/GU) ≈ r(CG/GC) ≈ r(GA/CU) < r(GG/CC) < r(GC/CG). The quantitative effect of molecular crowding on the individual NN pairs was estimated by subtracting parameters in the absence of a cosolute of the same cation concentration (37) (Table S3) from the obtained NN parameters in Table 1 and were shown in Table S4. As revealed by Table S4, some of the NN pairs were destabilized by the crowding, whereas others were minimally affected or conversely stabilized. This was likely due to the different states of hydration associated with each NN pair. Cosolutes disrupt the hydration network around nucleic acids and destabilize them (38). Each base pair exhibits variation among the helicities and geometries of donors and acceptors for hydrogen bonding, inducing different levels of hydration around the grooves for each NN pair (39). For four NN pairs [r(AA/UU), r(GU/CA), r(GC/CG) and r(GG/CC)], associated water molecules may be available to interact with cosolutes, which would result in the reduction in the stability of the pairs. However, for five pairs [r(AU/UA), r(UA/AU), r(CU/GA), r(GA/CU) and r(CG/GC)], water molecules may be buried in deeper grooves, making them unavailable to interact with cosolutes and causing a nominal decrease in stability. For, r(CA/GU) pair cosolutes may be assisting to maintain the hydration via H-bonding interaction, resulting in stabilization of the pair dominated by favorable enthalpy contribution.

**Table 1.** Nearest-neighbor parameters for RNA duplexes in the presence of 40 wt% PEG200 and 100 mM NaCl at 37°C^a^

Sequence	Δ*H*°_NN_ (kcal mol^−1^)	Δ*S*°_NN_ (cal mol^−1^ K^−1^)	Δ*G*°_37, NN_ (kcal mol^−1^)
r(AA/UU)	−10.0 ± 0.1	−30.4 ± 0.2	−0.57 ± 0.05
r(AU/UA)	−10.1 ± 0.1	−30.8 ± 0.1	−0.55 ± 0.03
r(UA/AU)	−11.1 ± 0.4	−31.5 ± 0.8	−1.33 ± 0.09
r(CA/GU)	−12.1 ± 0.3	−32.1 ± 0.6	−2.14 ± 0.08
r(GU/CA)	−10.7 ± 0.2	−28.7 ± 0.1	−1.80 ± 0.16
r(CU/GA)	−11.2 ± 0.3	−30.4 ± 0.3	−1.77 ± 0.17
r(GA/CU)	−11.7 ± 0.2	−30.5 ± 0.3	−2.24 ± 0.07
r(CG/GC)	−11.1 ± 0.5	−28.8 ± 1.1	−2.16 ± 0.16
r(GC/CG)	−13.8 ± 0.1	−34.6 ± 0.1	−3.07 ± 0.01
r(GG/CC)	−14.8 ± 0.1	−38.4 ± 0.1	−2.89 ± 0.06
Initiation	4.6 ± 2.0	−2.9 ± 6.1	5.50 ± 0.11
Per terminal AU	6.5 ± 0.1	18.2 ± 0.1	0.85 ± 0.92
Self-complementary	0	–1.4	0.43
Non-self-complementary	0	0	0

^a^Experiments were performed in 10 mM Na_2_HPO_4_,1 mM Na_2_EDTA, 100 mM NaCl and 40 wt% PEG200 at pH 7.0.

The effect of crowding was significant for helix initiation and terminal A–U pairs. Helix initiation and terminal A–U pairs were destabilized by 1.41 and 0.40 kcal mol^−1^, respectively, in the 40 wt% PEG200 solution (Table S4). This suggests that the formation of very short RNA duplexes under conditions of molecular crowding may be hindered due to the large unfavorable energy required for forming the first base pair in the duplex, while several NN pairs may be needed to compensate for this energy. The hydration around the terminal of a duplex is different from that of the stem as water molecules around the terminals are more accessible, resulting in greater disruption by the crowders. Therefore, the relative effect of crowders on NN pairs depends on the hydration around the particular NN pair.

The average differences in Δ*H*°, Δ*S*°, Δ*G*°_37_ and *T*_m_ between the predicted and measured values among the 45 studied RNA duplexes were 5.0%, 5.5%, 3.5% and 1.1°C, respectively (Table S5), which were well within the error limits reported in previous studies (22,28). Therefore, our derived parameters can be used to precisely predict the stability and thermodynamics of RNA duplex formation under physiological crowding condition.

### Development of generalized NN parameters for diverse crowding conditions

The molecular environments in cell vary spatiotemporally with the cell cycle, leading to different crowding densities (40). Moreover, cation concentration changes dynamically in different cellular compartments. Therefore, crowding conditions in different regions of a cell cannot be represented by a single cosolute at a particular cation concentration. Thus, to develop a generalized NN parameter set applicable to different cation and cosolute conditions, we separated each NN pair (Δ*G*°_NN_) into the individual contributions of the bulk interactions (Δ*G*°_NN, bulk_) and environmental factors (Δ*G*°_NN, env_) comprising of the energetic contribution of the cation (Δ*G*°_NN, cation_) and crowder (Δ*G*°_NN, crowder_). Therefore, each NN pair (Δ*G*°_NN_) under crowding conditions can be expressed as follows:


(5) $$\begin{eqnarray*} \Delta G{^\circ _{{\rm{NN}}}} &=& \Delta G{^\circ _{{\rm{NN}},{\rm{ bulk}}}} + \Delta G{^\circ _{{\rm{NN,env}}}} \\ \nonumber &=& \Delta G{^\circ _{{\rm{NN, bulk}}}} + \Delta G{^\circ _{{\rm{NN,cation}}}} + \Delta G{^\circ _{{\rm{NN,crowder}}}} \end{eqnarray*}$$

The bulk interactions consist of hydrogen bonding in base pairs, stacking interactions between neighboring base pairs, and conformational entropic penalty to bring two single strands into a helical structure (39). According to Equation 5, Δ*G*°_NN_ is equal to Δ*G*°_NN, bulk_ in the absence of contributions from Δ*G*°_NN, cation_ and Δ*G*°_NN, crowder_. In the absence of a crowder, Δ*G*°_NN_ showed exponential decay with increasing concentration of Na^+^ (Supplementary Figure S3). However, NN pairs with only A-U pairs deviated relatively more from the fitted line because of larger fluctuations in their reported Δ*G*°_NN_ values at different Na^+^ concentrations (Supplementary Figure S3) (37). Theoretically Δ*G*°_NN, bulk_ should be calculated by extrapolating the individual Δ*G*°_NN_ to zero Na^+^ concentration in the absence of a crowder, however, duplex formation without any cation is practically unrealistic. We found that stable oligomeric RNA duplexes with analyzable melting profile can be formed at minimum of 10 mM Na^+^ concentration (Supplementary Figure S4). Thus, Δ*G*°_NN_ values at 10 mM Na^+^ were approximated as Δ*G*°_NN, bulk_ for individual NN pairs and listed in Table 2, although numerical values at 10 mM Na^+^ did not change much compared to the values at zero Na^+^ concentration (Supplementary Figure S3). The Δ*G*°_NN, cation_ values can be obtained by subtracting Δ*G*°_NN, bulk_ from the reported Δ*G*°_NN_ for different cation concentrations in the absence of a crowder, as shown in Table 2, for 122 mM Na^+^ (37). Because Δ*G*°_NN_ for RNA duplexes is available only for Na^+^ (37), we can determine the Δ*G*°_NN, cation_ for different Na^+^ concentrations. However, for other cations such as K^+^ and Mg^2+^, some corrections are required to compensate for the differential effects from the Na^+^ parameters (see later sections).

**Table 2.** Parameters for Δ*G*°_37, NN, bulk_, Δ*G*°_37, NN, [122 mM Na_^+^_]_, Δ*G*°_37, NN, ev_ and Δ*G*°_37, NN, wa_ in 40 wt% PEG200 along with prefactors (*m*_cs_) for different cosolutes^a^

Sequence	Δ*G*°_37, NN, bulk_ (kcal mol^−1^)	Δ*G*°_37, NN, [122 mM Na_^+^_]_^b^ (kcal mol^−1^)	Δ*G*°_37, NN, ev [40 wt% PEG200]_^c^ (kcal mol^−1^)	Δ*G*°_37, NN, wa [40 wt% PEG200]_ (kcal mol^−1^)	*m*_PEG/2-ME/1,2 DME_ (kcal mol^−1^)	*m*_EG/Gly/1,3 PDO_ (kcal mol^−1^)
r(AA/UU)	−0.70	−0.00	−0.22	0.35	7.1	2.9
r(AU/UA)	−0.22	−0.30	−0.22	0.19	3.9	1.6
r(UA/AU)	−1.22	−0.08	−0.22	0.19	3.9	1.6
r(CA/GU)	−1.69	−0.09	−0.22	−0.14	−2.9	−1.2
r(GU/CA)	−1.73	−0.41	−0.22	0.56	11.4	4.7
r(CU/GA)	−1.52	−0.28	−0.22	0.25	5.1	2.1
r(GA/CU)	−2.06	−0.16	−0.22	0.20	4.1	1.7
r(CG/GC)	−2.00	−0.18	−0.22	0.24	4.9	2.0
r(GC/CG)	−2.72	−0.58	−0.22	0.45	9.2	3.8
r(GG/CC)	−2.92	−0.18	−0.22	0.43	8.8	3.6
Initiation	4.09	0.0^d^	−0.22	1.63	33.3	13.7
Per terminal AU	0.45	0.0^d^	NA^e^	0.40	8.2	3.4

^a^Correction factor for self-complementary sequences is the same (0.43 kcal mol^−1^) for all cosolutes because it is independent of the solution condition. ^b^Δ*G*°_37, NN, cat_ for any concentration of Na^+^ can be obtained from the Na^+^ dependency of individual NN pairs, as mentioned in Supplementary Figure S3. ^c^Averaged over all 45 sequences used in this study. ^d^Terminal pairs were assumed to be independent of Na^+^ concentration following the report by Huguet *et al.* (47) and thus, cation-induced stabilization was not added to terminal pairs. ^e^Excluded volume effect for terminal AU pairs was not considered to avoid overestimation of the effect, as it was already considered for initiation.

### Calculation of the excluded volume effect by the crowders

For Δ*G*°_NN, crowder_, individual contributions from the excluded volume effect (Δ*G*°_NN, ev_) and water activity (Δ*G*°_NN, wa_) should be quantified since those are the dominant physical and chemical factors, respectively, for the duplex stability in cosolute solutions.(25,27,41). By summing the contributions from the physicochemical properties, Δ*G*°_NN, crowder_ can be represented as follows:


(6) $$\begin{equation*}\Delta G{^\circ _{{\rm{NN,crowder}}}} = \Delta G{^\circ _{{\rm{NN, ev}}}} + \Delta G{^\circ _{{\rm{NN,wa}}}}\end{equation*}$$

Replacing Δ*G*°_NN, crowder_ in Equation 5 by Equation 6 results in the following relation:


(7) $$\begin{equation*}\Delta G{^\circ _{{\rm{NN}}}} = \Delta G{^\circ _{{\rm{NN, bulk}}}} + \Delta G{^\circ _{{\rm{NN,cation}}}} + \Delta G{^\circ _{{\rm{NN, ev}}}} + \Delta G{^\circ _{{\rm{NN,wa}}}}\end{equation*}$$

**Figure 2.**
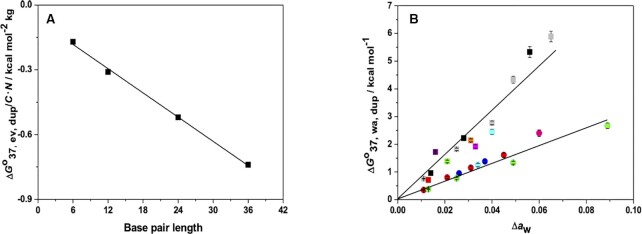
(**A**) Plot of excluded volume effect of RNA-PEG interaction against the base pair length of RNA duplexes. Data are provided in Table S7. (**B**) Plot of the contribution of water activity on the stability of GAUUACGCCUG against Δ*a*_w_ in EG (blue circles), Gly (red circles), 1,3 PDO (green circles), PEG200 (grey squares), 2-ME (green square), 1,2-DME (orange square), PEG400 (purple square), PEG600 (red square) at 100 mM NaCl and in EG (pink circle), 1,3-PDO (cyan circle), PEG200 (black squares), 2-ME (cyan square), 1,2-DME (magenta square) at 1 M NaCl. EG, Gly and 1,3-PDO belong to the same group (circles) whereas PEGs, 2-ME and 1,2-DME are in another group (squares). Data are presented in Table S8.

The volume excluded by crowders influences the stability of nucleic acids by shifting the equilibrium towards the formation of more compact structures with reduced volume (42). As an example, duplex formation is stabilized in the presence of larger biomolecules and cosolutes (43,44). The excluded volume effect is purely a physical interaction and has no dependence on the chemical nature of the cosolute or nucleic acid, supporting the additive nature of Equation 6. Excluded volume depends on length of the sequence as well as size of the cosolute. In the presence of significantly higher concentration of cosolute compared to nucleic acid, contribution of excluded volume to duplex stability (Δ*G*°_ev, dup_) was determined using the following equation: (45)


(8) $$\begin{equation*}\Delta G{^\circ _{{\rm{ev, dup}}}} = RT \bullet \rho \bullet {\rm{ }}\Delta V \bullet C\end{equation*}$$

where *R* is the gas constant in kcal mol^−1^ K^−1^, *T* is the temperature in kelvin (K), *ρ* is the density of water in kg L^−1^, Δ*V* is the change in the excluded volume between the nucleic acid and the cosolute upon duplex formation in L mol^−1^, and *C* is the molal concentration of the cosolute in mol kg^−1^ (45). Δ*V* for duplex formation can be represented as follows:


(9) $$\begin{equation*}\Delta V = {V_{{\rm{dup}}}}-2{V_{{\rm{ss}}}}\end{equation*}$$

where *V*_dup_ and *V*_ss_ are the volumes excluded by the duplex and single strands, respectively. *V* was determined from the following relation, considering RNAs as cylinders and cosolutes as polymeric chains (46);


(10) $$\begin{equation*}V = N \bullet {l^2} \bullet {N_A} \bullet k \bullet {\rm{ }}{10^{ - 27}}\end{equation*}$$

where *N* is the number of monomers present in the cosolute, *l* is the statistical segment length of the polymer in Å, *N*_A_ is the Avogadro's number (6.022 × 10^23^ mol^−1^), and *k* is a geometrical factor derived from the length and radius of the RNA in Å (Table S6). The factor 10^−27^ is used for the conversation of Å^3^ to L. Using the parameters listed in Table S6, we calculated *V*/*N* for different lengths of RNA in PEG using Equation 10 (Table S6). From the values of *V*/*N* in Table S6, Δ*V*/*N* and Δ*G*°_37, ev, dup_/*C*•*N* were calculated using Equations 9 and 8, respectively, for RNA-PEG interactions for different lengths of RNA duplexes (Table S7). Δ*G*°_37, ev, dup_/*C*•*N* decreased as the length of the RNA increased (Figure 2A), and showed a good linear correlation with the length of the duplex, producing the following equation:


(11) $$\begin{equation*}\Delta G{^\circ _{37,{\rm{ ev, dup}}}}/C \bullet N = - 0.019 \bullet n - 0.070\end{equation*}$$

where *n* denotes the number of base pairs. The strong linear correlation (correlation coefficient of 0.999) suggested that Equation 11 can be used to calculate Δ*G*°_37, ev, dup_ for any given length of RNA duplex and cosolute concentration. Assuming that all NN pairs have the same volume effect, the contribution of the excluded volume effect for each NN pair and initiation factor (Δ*G*°_37, NN, ev_) was determined by the relation below (Equation 12). The values for 40 wt% PEG200 are shown in Table 2.


(12) $$\begin{equation*}\Delta G{^\circ _{37,{\rm{ NN, ev}}}} = \Delta G{^\circ _{37,{\rm{ev, dup}}}}/n\end{equation*}$$

### Determination of the contribution of water activity of the cosolute solutions

To explore the relationship between the RNA duplex stability and the water activity of the solution, we measured the stability of a test RNA sequence in solutions with different water activities as obtained by different cosolutes at varying concentrations (Table S8). We selected GAUUACGCCUG as a test sequence because it contained all 10 NN base pairs with a single occurrence, which eliminated bias toward any particular NN pair. The contribution of water activity to the duplex (Δ*G*°_37, wa, dup_) for the test sequence in different cosolute solutions was calculated by subtracting the stability in the absence of cosolute (equivalent to Δ*G*°_37, bulk, dup_ + Δ*G*°_37, cation, dup_) and Δ*G*°_37, ev, dup_ from the measured Δ*G*°_37_ (Table S8). For example, in 10 wt% EG solution at 100 mM NaCl, Δ*G*°_37, wa, dup_ for GAUUACGCCUG was calculated to be 0.95 kcal mol^−1^ by as follows: Δ*G*°_37, wa, dup_ = Δ*G*°_37_ − Δ*G*°_37, no cosolute_ - Δ*G*°_37, ev, dup_, where Δ*G*°_37_, Δ*G*°_37, no cosolute_ and Δ*G*°_37, ev, dup_ were −13.6, −14.1 and −0.45 kcal mol^−1^, respectively (Table S8). The plot of Δ*G*°_37, wa, dup_ against the changes in water activity, Δ*a*_w_ (Δ*a*_w_ =*a*_no cosolute_ − *a*_cosolute_) revealed two different linear correlations depending on the chemical structures of the cosolutes (Figure 2B). Cosolutes lacking vicinal hydroxyl groups, such as PEGs, 2-methoxy ethanol (2-ME), and 1,2-dimethoxyethane (1,2-DME), showed a higher order of destabilization. On the other hand, the extent of destabilization was reduced in the presence of ethylene glycol (EG), glycerol (Gly) and 1,3-propanediol (1,3-PDO) with hydroxyl groups in the vicinity. The destabilization of the duplex by the addition of a cosolute was assumed to be due to the disruption of the ordered water network around the duplex (38). However, the cosolutes containing hydroxyl groups in close vicinity, such as EG, Gly and 1,3-PDO, form H-bonds with the water molecules surrounding the duplex, which can help to maintain the ordered water structure around the duplex even under crowding conditions, which reduces destabilization. As shown in Figure 2B, the lowest destabilization was observed in the 1,3-PDO solutions (green circles). This is due to the suitable positioning of the two hydroxyl groups in 1,3-PDO for the H-bonding interaction with water molecules (48), resulting in higher stabilities dominated by favorable enthalpy contributions as compared to the solution without any cosolute (Table S8). Interestingly, we observed the same correlation for cosolutes in both 1 M and 100 mM NaCl solutions, suggesting that Δ*G*°_37, wa, dup_ is independent of the ionic concentration of the solution, thus validating the additive nature of Equation 7. Because we found a linear relationship between Δ*G*°_37, wa, dup,_ and Δ*a*_w_, it is reasonable to assume that each of the 10 NN base pairs and terminal parameters (Δ*G*°_37, NN, wa_) is also linearly correlated with the water activity of the solution by the following relation:


(13) $$\begin{equation*}\Delta G{^\circ _{37,{\rm{ NN, wa}}}} = {m_{{\rm{cs}}}} \bullet \Delta {a_{\rm{w}}}\end{equation*}$$

where *m*_cs_ is a prefactor for cosolutes, which is equivalent to the energy parameter for NN base pairs in the presence of a cosolute, dependent on the individual NN base pair and cosolute. Subtracting the contributions of bulk (Δ*G*°_37, NN, bulk_), cation (Δ*G*°_37, NN, [122 mM Na_^+^_]_) and excluded volume effect (Δ*G*°_37, NN, ev_) from the Δ*G*°_37, NN_ parameters of Table 1 provided Δ*G*°_37, NN, wa_ for 40 wt% PEG200, as shown in Table 2. The *m*_cs_ values for PEG (*m*_PEG_) were calculated from the Δ*G*°_37, NN, wa [40 wt% PEG200]_ values using Equation 13 (Table 2). The *m*_cs_ values of the other cosolutes were calculated as follows:


(14) $$\begin{equation*}{m_{{\rm{cs}}}} = {m_{{\rm{PEG}}}} \bullet \left( {{S_{{\rm{CS}}}}/{S_{{\rm{PEG}}}}} \right)\end{equation*}$$

where *S*_CS_ and *S*_PEG_ are the slopes for the cosolute of interest and PEG, respectively, in the Δ*G*°_wa, dup_ vs. Δ*a*_w_ plot (Figure 2B). Δ*G*°_37, NN, wa_ for different cosolutes can be calculated by multiplying *m*_cs_ (Table 2) and Δ*a*_w_. The water activities (*a*_w_) in different cosolute solutions are available in the literature or can be measured by osmometry, as mentioned in the Supplementary data. Therefore, the stability of RNA duplexes in different crowded environments can be predicted from their base sequences, using the universal parameters listed in Table 2.

### Validation of the generalized NN parameters for different *in vitro* crowded conditions

Although the NN parameters were established from the experimental data of Na^+^-containing solutions, the intracellular environment contained K^+^ as the major monovalent cation (49,50). Therefore, considering the utility of the parameters for intracellular reactions, we verified the suitability of our parameters for the prediction of RNA stability in 100 mM KCl under the same crowding condition with 16 RNA duplexes selected from Table S2 differing in length and base composition. The measured Δ*G*°_37_ and *T*_m_ values indicated that, under the molecular crowding induced by 40 wt% PEG200, stabilities of RNA duplexes in Na^+^ were higher than those in K^+^ with the average difference of 12.1%, (Table S9). This could be as a result of the different binding modes of the two cations with the RNA (51); K^+^ interacts with the major grooves, whereas Na^+^ preferentially binds to highly charged phosphates, leading to more efficient charge screening. These differences were found significant when the dielectric constant was dramatically dropped, such as in 40 wt% PEG200 where dielectricity reduced to almost 50% compared to the non-crowding solution (Table S10), favoring electrostatic interaction between negatively charged phosphates and Na^+^. However, in the absence of cosolute and in 40 wt% EG, where dielectricity did not decrease much, RNA duplexes showed only a marginal increase in their stability in the presence of Na^+^ compared with K^+^ with an average differences of 3.1% and 4.2%, respectively (Tables S11 and S12). We investigated the quantitative relationship of RNA stability between K^+^ and Na^+^ in 40 wt% PEG200 to obtain duplex stability in the presence of K^+^ from their values in the Na^+^ solution. We observed a good linear relationship between the measured stability in K^+^ and the predicted stability in the same concentration of Na^+^ solution using our derived parameters (Supplementary Figure S5A) to produce following equation:


(15) $$\begin{eqnarray*}\Delta G{^\circ _{37}}\left( {{\rm{measured\, in\, }}{{\rm{K}}^ + }} \right) &=& 0.93{\rm{ }}\Delta G{^\circ _{37}}\left( {{\rm{predicted\, in\, N}}{{\rm{a}}^ + }} \right)\nonumber\\ && +\, 0.45\end{eqnarray*}$$

The slope of the equation is defined as the ratio of the change in Δ*G*°_37_ values between the K^+^ and Na^+^ solutions. The value of 0.93 indicated that Δ*G*°_37_ decreased by 7% in the K^+^ condition compared to the Na^+^ solution because of the lower charge screening of RNA duplexes in the K^+^ medium. Henning-Knechtel et al. reported a 6% lesser charge neutralization by K^+^ than by Na^+^ for HIV-1 TAR RNA hairpins, as determined from molecular dynamics simulations (51). The intercept of the linear equation indicates the excess energy contribution in K^+^ medium when Δ*G*°_37_ (Na^+^) is zero. This excess energy contribution is mainly affected by structural factors, such as duplex terminals. Positive intercept suggested that, under crowding conditions, K^+^ destabilizes the duplex terminals on average by 0.45 kcal mol^−1^ compared to Na^+^. Using Equation 15, the calculated Δ*G*°_37_ of the 16 duplexes in the K^+^ solution showed an average difference of only 4.3% from the measured values (Table S13), suggesting that, without calculating the NN parameters in the KCl condition, our derived parameters in 100 mM NaCl solution can provide an accurate estimation of RNA stability in 100 mM KCl following a simple linear relation.

**Figure 3.**
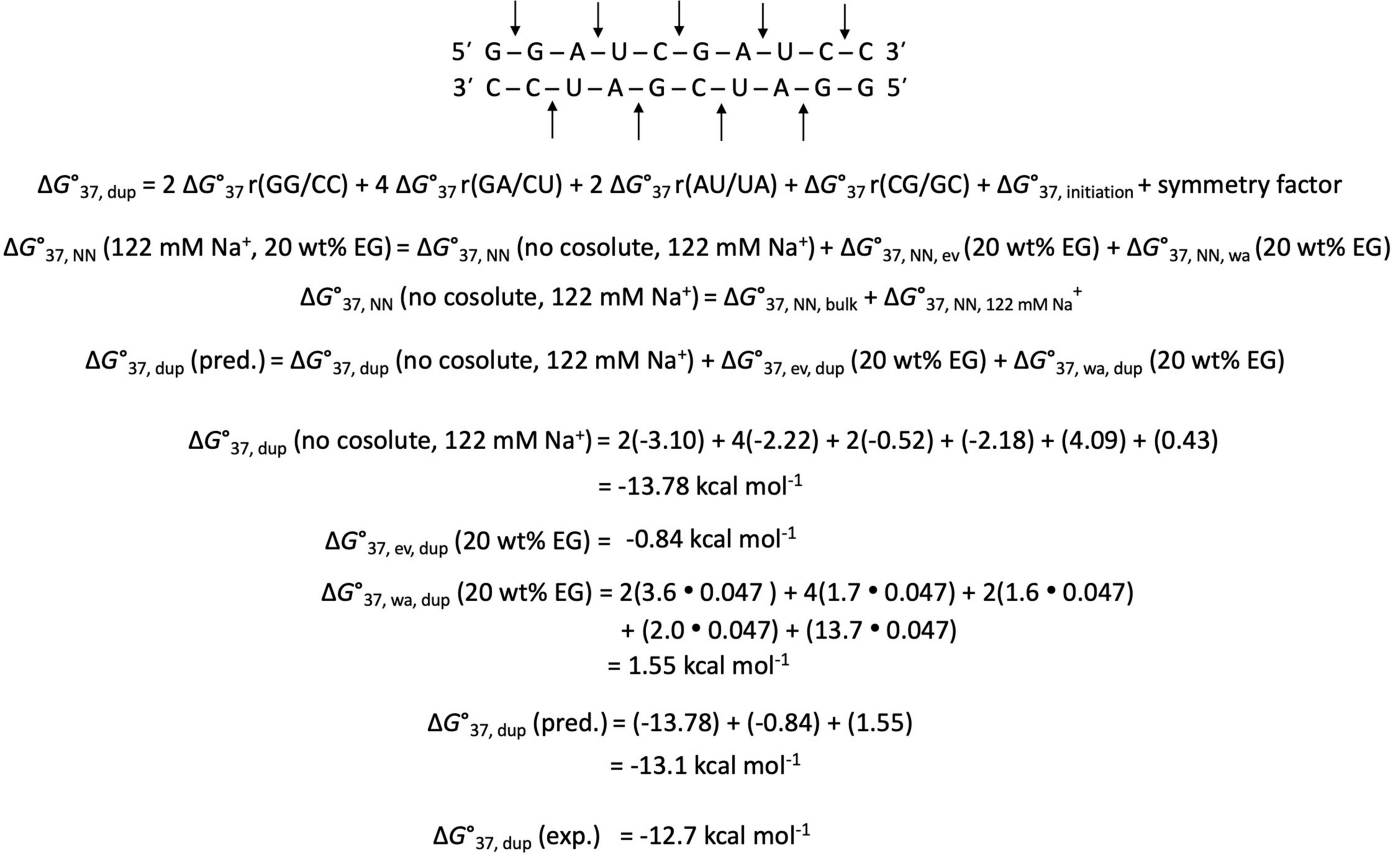
Prediction of Δ*G*°_37_ of sequence GGAUCGAUCC in solution containing 122 mM Na^+^ and 20 wt% EG. Each arrow represents one NN set. Δ*G*°_37, NN, bulk_ and Δ*G*°_37, NN, 122 mM Na_^+^ values from Table 2 were used. Δ*G*°_37, ev, dup_ was calculated using Equation 11 and the Δ*G*°_37, wa,_ duplex was determined using the *m*_cs_ values obtained from Table 2, using Equation 13. Δ*a*_w_ value of the solution was 0.047. This duplex is self-complementary, with symmetry factor of 0.43 kcal mol^−1^.

We verified the applicability of the generalized parameters for different crowding conditions using a large number of RNA sequences with different NN compositions and lengths. We used the parameters in Table 2 to predict RNA duplex stability at different concentrations of a cosolute, different types of cosolutes, and different NaCl concentrations (Table S14). The reported stabilities of 38 RNA duplexes (28) in 20 vol% PEG200 at 1 M NaCl were also predicted (Table S14). Although NN parameters reported by Adams and Znosko predicted the measured thermodynamic parameters for 38 sequences with accuracy (28), unlike the current generalized parameters, those parameters are only applicable for a specific crowding condition. The calculation of Δ*G*°_37_ for a sequence in a particular cosolute solution using generalized parameters is illustrated in Figure 3. The average difference between the predicted and measured Δ*G*°_37_ values for the 52 sequences listed in Table S14 was 7.3%. Therefore, these results indicate that the generalized parameters can predict RNA duplex stability under different NaCl conditions and in various cosolutes, differing largely in their molecular weights, with significant accuracy.

### Stability prediction of RNA structures in local intracellular conditions

For the established parameters to predict RNA duplex stabilities in cells, which contain monovalent cations such as Na^+^ and K^+^, and small amounts of divalent cations such as Mg^2+^, the approximation of Δ*G*°_NN, cation_ is needed because the database of Δ*G*°_NN, cation_ values was available for a single component of the cation. To verify the applicability of the derived parameters under intracellular cation conditions, we measured thermodynamic parameters for the same 16 RNA duplexes in a buffer solution containing the exact intracellular cation composition (140 mM K^+^, 10 mM Na^+^, 0.5 mM Mg^2+^ and 0.0001 mM Ca^2+^) (49,50) under 40 wt% PEG200 at a pH of 7.2. The measured values were in agreement with the thermodynamic parameters and *T*_m_ of the duplexes measured in 122 mM Na^+^ with 40 wt% PEG200 (Table S15), indicating that the combined effect of monovalent and divalent cations on the duplex stability at the intracellular concentration is similar to that of the Na^+^ condition used for determining the NN parameters. When we plotted the measured Δ*G*°_37_ in the 40 wt% PEG200 with the intracellular cation concentration against the corresponding predicted values in the Na^+^ solution with 40 wt% PEG200 (Figure S5B), we obtained a straight line with the following equation.


(16) $$\begin{eqnarray*} && \Delta G{^\circ _{37}}\left( {{\rm{measured\, in\, intracellular\, cation\, conc}}{\rm{.}}} \right)\nonumber\\ && \quad = 0.92{\rm{ }}\Delta G{^\circ _{37}}\left( {{\rm{predicted\, in\, N}}{{\rm{a}}^ + }} \right) - 0.12\end{eqnarray*}$$

Equation 16 revealed a slope of 0.92, similar to the slope of Equation 15, suggesting a similar extent of charge neutralization of RNAs by intracellular cation composition and 122 mM K^+^. However, contrary to Equation 15, the linear plot produced a slightly negative intercept of 0.12 kcal mol^−1^, suggesting that duplex terminals were stabilized by intracellular cation concentration as Mg^2+^ can bind to the N7 atom of the exposed purine bases at terminals (52). The combined effect of charge neutralization and terminal stabilization by the intracellular cation composition resulted in a similar stabilization to the Na^+^ condition used for determining the NN parameters, as evident from the data in Table S15. As a result, the prediction errors showed average differences in Δ*H*°, Δ*S*°, Δ*G*°_37_ and *T*_m_ of 7.4%, 7.7%, 6.7% and 1.9°C, respectively (Table S16), validating the applicability of the derived NN parameters for intracellular cation conditions. Therefore, molecular crowding conditions with 122 mM Na^+^ should be ideal as a model solution for studying physiological crowding in cells for physicochemical measurements.

According to the approximation of physiological cation condition as above, we demonstrated stability prediction of RNA duplexes in cells. It should be remembered that experimental data on nucleic acid stability in cells are limited, owing to the complexity of measurements and sophisticated instrumentation, as well as the crowding condition inside a cell is dynamic and heterogeneous. Thus, we can validate only few available data for RNA structures to demonstrate the applications of the established NN parameters for in-cell stability prediction. Here, we applied to predict RNA duplexes under nucleolus-like conditions in cells. In the report by Nott et al. (53), ΔΔ*G*°_25_ (difference in Δ*G*°_25_ between inside and outside the nucleolus) for two RNA duplexes, (ACUG)_3_ and (ACUG)_6_ were measured to be 3.0 and 3.3 kcal mol^−1^ at 25°C, respectively. Since, these destabilizations were mainly due to the crowding condition inside the nucleolus, it can be considered as Δ*G*°_[crowder]_ at 25°C. We used our generalized Δ*G*° parameters adjusted for 25°C using Δ*H*° and Δ*S*° values (see Table S17) to determine the cosolute condition that imposes similar destabilizations, as reported for the two duplexes. We found that using our generalized parameters the measured destabilizations were matching with the predicted Δ*G*°_25, [crowder]_ in 40∼50 wt% PEG200 solution (see Table S18), which is the same condition predicted using our DNA NN parameters for mimicking nucleolus (27). This result suggested the reliability of our newly derived RNA generalized parameters in predicting diverse crowding conditions both *in vitro* and intracellular environments.

Moreover, we demonstrated the prediction of hairpin RNA (Im-4U), mutated from *Salmonella* fourU-type RNA thermometer (4U) hairpin sequence located in 5′-UTR of the agsA gene (54). In Im-4U, G–U wobble base pairs, a hairpin loop, and an internal loop exist (Supplementary Figure S6). For G–U wobble base pairs, we estimated the stability of NN parameters including G-U pair according to the rules of the changes in NN pairs containing A–U pair because both base pairs have two hydrogen bonds, that are approximated to have same energy contribution of A–U pair (Table S19) (55). For loops, as the destabilization effect of the hairpin tetraloop was reduced by 58% in 40 wt% PEG200 (Table S20) (56), we formulated an empirical relation for loop stability in the cosolute solutions (Δ*G*°_37, loop, cosolute_) from their stability in the absence of cosolute (Δ*G*°_37, loop, no cosolute_) and change in the water activity of cosolute solution (Δ*a*_w_) as follows:


(17) $$\begin{equation*}\Delta G{^\circ _{37,{\rm{ loop, cosolute}}}} = \Delta G{^\circ _{37,{\rm{ loop, no cosolute}}}} \bullet \left( {1 - 11.8{\rm{ }} \bullet {\rm{ }}\Delta {a_{\rm w}}} \right)\end{equation*}$$

The details of these approximations were described in Supplementary Data. The excluded volume effect for the hairpin was determined following the same procedure as described earlier; only parameters for the hairpin were different from duplex (Table S6). From these parameters and relationship (Equation 17), the stability of the Im-4U in 40 wt% PEG200 at 100 mM NaCl was estimated to be 1.1 kcal mol^−1^ (Supplementary Figure S6). The predicted value complied satisfactorily with the measured stability of 0.4 kcal mol^−1^, compared to the value of –2.7 kcal mol^−1^, predicted using classical NN parameters. The positive Δ*G*°_37_ of the predicted value indicates that destabilization by the loop was estimated well by the developed parameters, as other stem regions should stabilize the structure that negatively contributes to Δ*G*°_37_ (Table 1). Thus, this result suggests the dependability of the assumptions for the stability of G–U wobble pairs and loops under crowded condition.

Finally, we used the expanded parameters to estimate the crowding conditions in nucleus and cytosol. Δ*G*°_37_ of Im-4U in the nucleus and cytosol of live HeLa cells were reported to be –1.7 and –2.6 kcal mol^−1^, respectively (54). The predicted Δ*G*°_37_ of Im-4U in PEG200 showed a significant destabilizing effect on the structure (1.1 kcal mol^−1^ in 40 wt% PEG200) and did not match the reported Δ*G*°_37_ inside HeLa cells. Thus, we used less destabilizing cosolutes (EG, Gly, and 1,3 PDO) to estimate the intracellular crowding *in vitro*. We identified that the Δ*G*°_37_ of Im-4U in the nucleus and cytosol of HeLa cells were successfully predicted to be –1.9 and –2.7 kcal mol^−1^, respectively, using our parameters in 30 wt% and 40 wt% 1,3-PDO conditions at 100 mM NaCl (Table S21). These solution conditions were different from that of the nucleolus, as mentioned above, indicating that our prediction is versatile in different localized cellular regions. In contrast, it should be noted that the experimental Δ*G*°_37_ exhibited a wide distribution of about 2.0 kcal mol^−1^ in the measured values in the nucleus and cytosol (54). This wide distribution suggests that some Im-4U strands further localized in environments such as liquid-liquid separated regions in the cytosol and nucleus, which were significantly different from other regions, and gained more stability in the cytosol than in the nucleus or vice versa. Therefore, our determined parameters will provide accurate stability of the secondary RNA structures in cells when the molecular environments of cellular compartments are well resolved; this requires high-resolution stability data for RNA structures in individual localized regions of a cell.

## CONCLUSION AND GUIDE FOR USERS

We developed prediction parameters of *in vitro* and in-cell stability of RNA duplex formation based on the NN model available in diverged crowding conditions. Our method proposed a general approach to obtain the NN parameters for different types of commonly used cosolutes, which is a sum of four separated NN parameters: 1) Δ*G*°_37, NN, bulk_ from Table 2, 2) Δ*G*°_37, NN, Na_^+^ from Table 2, 3) Δ*G*°_37,NN, ev_ from Table 2 calculated using Equations 12, and 4) Δ*G*°_37,NN, wa_ determined from the *m*_CS_ values in Table 2 using Equation 13. For example, by changing Δ*G*°_37,NN, ev_ and Δ*G*°_37,NN, wa_ terms, specific NN parameters for the nucleolus condition using 40 or 50 wt% PEG200 in 100 mM NaCl solution and the cytosol condition using 40 wt% 1,3 PDO with 100 mM NaCl can be obtained. The users can freely arrange the parameters for predictions in their specific conditions containing different concentrations of Na^+^ and crowders using these table and equations (as demonstrated in Figure 3).

A prediction of the thermodynamic stability of RNA duplexes under diverse crowding conditions is a basis to determine RNA folded structures in spatiotemporal area in cells. Further collection of parameters of various structural motifs like bulges, mismatches, and dangling ends will enable to predict large RNA structures *in vitro* and in cells. Our approach should be beneficial for not only investigating biological processes but also for designing RNA structures for RNA interference, antisense oligonucleotide therapy, and mRNA vaccines, which work efficiently in specific environments of different cells. Moreover, our method can provide the information of in-cell secondary structures which is currently unknown to develop RNA targeting drugs for RNA viruses including SARS-CoV-2.

## DATA AVAILABILITY

All relevant data are included in the paper and/or its supplementary data.

## Supplementary Material

gkad020_Supplemental_File
