# Supplementary material for: Nearest-neighbor parameters for the prediction of RNA duplex stability in diverse in vitro and cellular-like crowding conditions
Source: Nucleic Acids Res. 2023 Jan 31;51(9):4101–11. doi: 10.1093/nar/gkad020 (PMC10201447; doi:10.1093/nar/gkad020)
Supplement: gkad020_Supplemental_File [file gkad020_supplemental_file.pdf]

# Supporting Information

## Nearest-neighbor parameters for the prediction of RNA duplex stability in diverse *in vitro* and cellular-like crowding conditions

Saptarshi Ghosh,[1] Shuntaro Takahashi,[1] Dipanwita Banerjee,[1] Tatsuya Ohyama,[1] Tamaki Endoh,[1] Hisae Tateishi-Karimata,[1] and Naoki Sugimoto[1,2,]*

[1]Frontier Institute for Biomolecular Engineering Research (FIBER), [2]Graduate School of Frontiers of Innovative Research in Science and Technology (FIRST), Konan University, 7-1-20 Minatojima-minamimachi, Kobe 650-0047, Japan

**Contents:**

1. Supporting Method
2. Table S1, RNA sequences with nearest-neighbor frequencies
3. Table S2, Thermodynamic parameters of RNA duplexes measured in crowding condition and predicted parameters in the absence of cosolute
4. Table S3, Nearest-neighbor parameters in the absence of cosolute in 100 mM NaCl at 37 °C
5. Table S4, Stability contribution of 40 wt% PEG200 on the individual nearest-neighbor base pairs
6. Table S5, Measured and predicted thermodynamic parameters for RNA duplex formation with 40 wt% PEG200 and 100 mM NaCl in 10 mM phosphate buffer (pH 7.0)
7. Table S6, Parameters for RNA-PEG excluded volume calculations
8. Table S7, Excluded volume and its energy contribution for RNA duplexes with PEG
9. Table S8, Thermodynamic parameters of GAUUACGCCUG in different cosolute solutions and the water activity values of the solutions
10. Table S9, Measured thermodynamic parameters of duplexes in NaCl and KCl with 40 wt% PEG200
11. Table S10, Measured thermodynamic parameters of duplexes in NaCl and KCl without cosolute
12. Table S11, Dielectric constants of the media in the absence and presence of cosolutes
13. Table S12, Measured thermodynamic parameters of duplexes in NaCl and KCl with 40 wt% EG
14. Table S13, Measured and calculated $\Delta G°_{37}$ of RNA duplexes in 100 mM KCl with 40 wt% PEG200
15. Table S14, Measured and predicted $\Delta G°_{37}$ of RNA duplexes in different cosolutes and NaCl concentrations
16. Table S15, Measured thermodynamic parameters of RNA duplexes in NaCl and exact intracellular cation composition with 40 wt% PEG200
17. Table S16, Measured thermodynamic parameters of RNA duplexes in exact intracellular cation composition and predicted parameters in 100 mM NaCl with 40 wt% PEG200

18. Table S17, Nearest-neighbor parameters for $\Delta G°$ in the presence of PEG200 at 25 °C
19. Table S18, Calculated $\Delta G°_{25, [crowder]}$ for $(ACUG)_3$ and $(ACUG)_6$ in various PEG200 concentrations
20. Table S19, Estimated Nearest-neighbor parameters including G-U wobble base pairs for Im-4U
21. Table S20, $\Delta G°_{37}$ of a tetraloop in the absence and presence of PEG200
22. Table S21, Predicted $\Delta G°_{37}$ of RNA hairpin Im-4U in different cosolutes with $\Delta a_w$ values
23. Figure S1, CD spectra of sequences in dilute and crowding condition
24. Figure S2, Renaturation and denaturation curves for some representative RNA oligonucleotides in crowding condition
25. Figure S3, Variation of $\Delta G°_{37, NN}$ with $Na^+$ concentration in the absence of cosolute
26. Figure S4, Melting profiles of RNA duplexes with varying concentration of NaCl
27. Figure S5, Plot of $\Delta G°_{37}$ for RNA duplexes measured in KCl and exact intracellular cation concentration against predicted $\Delta G°_{37}$ by NaCl parameters under 40 wt% PEG200
28. Figure S6, Secondary structure and stability prediction of RNA hairpin Im-4U

## Supporting Method

## Energetic estimation of nearest-neighbor (NN) parameters for G-U base pair and loop contribution

For G-U wobble base pairs, we estimated the stability of NN parameters including G-U pair according to the rules of the changes in NN pairs containing A-U pair because both base pairs have two hydrogen bonds, that are approximated to have same energy contribution of terminal A-U pair (Table S19) (1). For example, $\Delta G°_{37, \text{NN, no cosolute}}$ value for r(GA/UU) in 100 mM NaCl was determined to be -0.96 kcal mol$^{-1}$ from its value of -1.27 kcal mol$^{-1}$ in 1 M NaCl, applying the same extent of reduction in the stability observed for r(AA/UU) in 100 mM NaCl from its value in 1 M NaCl. The $m_{\text{cs}}$ values for NN pairs containing G-U base pairs in different cosolutes were considered to be same as determined for NN pairs having A-U pair (Table S19). Empirical relationships were proposed for the stability of different types of loop structures in varying concentrations of $Na^+$ and $Mg^{2+}$, although negligible cation dependency was observed for shorter loops (2). A previous study suggested that the destabilization effect of the hairpin tetraloop was reduced by 58% in 40 wt% PEG200 (Table S20) (3). Based on this information and as the stability of the loop changes with changing the water activity of the solution, we formulated an empirical relation for loop stability in the cosolute solutions ($\Delta G°_{37, \text{loop, cosolute}}$) from their stability in the absence of cosolute ($\Delta G°_{37, \text{loop, no cosolute}}$) and change in the water activity of cosolute solution ($\Delta a_w$) as follows:

$$\Delta G°_{37, \text{loop, cosolute}} = \Delta G°_{37, \text{loop, no cosolute}} \cdot (1- 11.8 \cdot \Delta a_w)$$

$\Delta a_w$ is 0.049 for 40 wt% PEG200. The factor 11.8 was determined to match the extent of the reduction in the measured stability of a tetraloop in 40 wt% PEG200 (Table S20).

**Table S1.** RNA sequences with nearest-neighbor frequencies[a]

| | | Nearest-neighbor combinations present in duplex | | | | | | | | | |
|---|---|---|---|---|---|---|---|---|---|---|---|
| No. | Sequence[b] | AA UU | AU UA | UA AU | CU GA | CA GU | GU CA | GA CU | CG GC | GC CG | GG CC |
| NS1 | CAUGCC | | 1 | | | 2 | | | | 1 | 1 |
| NS2 | CUAGGC | | | 1 | 2 | | | | | 1 | 1 |
| NS3 | CAGCGG | | | | 1 | 1 | | | 1 | 1 | 1 |
| NS4 | CUACGC | | | 1 | 1 | | 1 | | 1 | 1 | |
| NS5 | GAACUCC | 1 | | | 1 | | 1 | 2 | | | 1 |
| NS6 | GGCUGUCC | | | | | | | | | | |
| NS7a | GGCUGUUC | 1 | | | 1 | 1 | 1 | 1 | | 1 | 1 |
| NS7b | GGUUCUGC | 1 | | | 1 | 1 | 1 | 1 | | 1 | 1 |
| NS8a | AGCUGUCU | | | | 3 | 1 | 1 | 1 | | 1 | |
| NS8b | AGUCUGCU | | | | 3 | 1 | 1 | 1 | | 1 | |
| NS9a | GGCAGUUC | 1 | | | 1 | 1 | 1 | 1 | | 1 | 1 |
| NS9b | GGUUCAGC | 1 | | | 1 | 1 | 1 | 1 | | 1 | 1 |
| NS10a | CGCUGUCG | | | | 1 | 1 | 1 | 1 | 2 | 1 | |
| NS10b | CGUCUGCG | | | | 1 | 1 | 1 | 1 | 2 | 1 | |
| NS11a | UGCUGUCA | | | | 1 | 3 | 1 | 1 | | 1 | |
| NS11b | UGUCUGCA | | | | 1 | 3 | 1 | 1 | | 1 | |
| NS12a | UAUGAGGA | | 1 | 1 | 1 | 1 | | 2 | | | 1 |
| NS12b | UAGAUGGA | | 1 | 1 | 1 | 1 | | 2 | | | 1 |
| NS13a | GUGCCGAG | | | | 1 | 1 | 1 | 1 | 1 | 1 | 1 |
| NS13b | GCCGAGUG | | | | 1 | 1 | 1 | 1 | 1 | 1 | 1 |
| NS14a | CGCUGUAG | | | 1 | 2 | 1 | 1 | | 1 | 1 | |
| NS14b | CGUGCUAG | | | 1 | 2 | 1 | 1 | | 1 | 1 | |
| NS15a | AUUGGAUACAAA | 3 | 2 | 1 | | 2 | 1 | 1 | | | 1 |
| NS15b | AUACAUUGGAAA | 3 | 2 | 1 | | 2 | 1 | 1 | | | 1 |
| NS16 | GGCUCAAUUGAC | 2 | 1 | | 1 | 2 | 1 | 2 | | 1 | 1 |
| S1 | UACAUGUA | | 1 | 2 | | 2 | 2 | | | | |
| S2 | GGUAUACC | | 1 | 2 | | | 2 | | | | 2 |
| S3 | GGUUAACC | 2 | | 1 | | | 2 | | | | 2 |
| S4 | CGAAUUCG | 2 | 1 | | | | | 2 | 2 | | |
| S5 | GCUUAAGC | 2 | 1 | | 2 | | | | | 2 | |
| S6 | CGUAUACG | | 1 | 2 | | | 2 | | 2 | | |
| S7 | CGCAUGCG | | 1 | | | 2 | | | 2 | 2 | |
| S8 | CGGUACCG | | | 1 | | | 2 | | 2 | | 2 |
| S9a | CAAGCUUG | 2 | | | 2 | 2 | | | | 1 | |
| S9b | CUUGCAAG | 2 | | | 2 | 2 | | | | 1 | |
| S10a | GAACGUUC | 2 | | | | | 2 | 2 | 1 | | |
| S10b | GUUCGAAC | 2 | | | | | 2 | 2 | 1 | | |
| S11a | GAUCCGGAUC | | 2 | | | | | 4 | 1 | | 2 |
| S11b | GGAUCGAUCC | | 2 | | | | | 4 | 1 | | 2 |
| S12a | AUGAGCUCAU | | 2 | | 2 | | 2 | 2 | | 1 | |

| | | | | | | | | | | | |
|---|---|---|---|---|---|---|---|---|---|---|---|
| S12b | AUCAGCUGAU | | 2 | | 2 | | 2 | 2 | | 1 | |
| S13 | UUACGCGUAA | 2 | | 2 | 2 | | 2 | | 2 | 1 | |
| S14 | AUCGCUAGCGAU | | 2 | 1 | | | | 2 | 2 | 2 | |
| S15a | CAUAGGCCUAUG | | 2 | 2 | 2 | | 2 | | | 1 | 2 |
| S15b | CUAUGGCCAUAG | | 2 | 2 | 2 | | 2 | | | 1 | 2 |

[a]Total frequencies of the NN parameters are as follows: r(AA/UU) = 29, r(AU/UA) = 27, r(UA/AU) = 24, r(CA/GU) = 46, r(GU/CA) = 36, r(CU/GA) = 45, r(GA/CU) = 43, r(CG/GC) = 26, r(GC/CG) = 31, and r(GG/CC) = 33. [b]RNA duplex consists of denoted RNA strand (5’ → 3’) and complementary RNA strand.

**Table S2.** Thermodynamic parameters for RNA duplexes measured in the crowding condition and predicted in the absence of cosolute

| | | Measured in the presence of 40 wt% PEG200[b] | | | | Predicted in the absence of cosolute[c] | | Difference between with and without cosolute | |
|---|---|---|---|---|---|---|---|---|---|
| No. | Sequence[a] | $\Delta H°$ (kcal mol$^{-1}$) | $T\Delta S°$ (kcal mol$^{-1}$) | $\Delta G°_{37}$ (kcal mol$^{-1}$) | $T_m$[d] (°C) | $\Delta G°_{37}$ (kcal mol$^{-1}$) | $T_m$[d] (°C) | $\Delta\Delta G°_{37}$ (kcal mol$^{-1}$) | $\Delta T_m$ (°C) |
| NS1 | CAUGCC | -56.5 ± 1.9 | -50.9 ± 1.7 | -5.6 ± 0.3 | 31.0 | -6.4 | 35.2 | 0.8 | -4.2 |
| NS2 | CUAGGC | -57.5 ± 1.9 | -51.4 ± 1.7 | -6.1 ± 0.3 | 34.3 | -7.2 | 39.8 | 1.1 | -5.5 |
| NS3 | CAGCGG | -65.6 ± 3.0 | -59.2 ± 2.7 | -6.4 ± 0.4 | 36.4 | -8.1 | 44.4 | 1.7 | -8.0 |
| NS4 | CUACGC | -62.8 ± 4.7 | -58.1 ± 4.4 | -4.7 ± 0.6 | 27.8 | -6.6 | 37.2 | 1.9 | -9.4 |
| NS5 | GAACUCC | -71.8 ± 2.3 | -65.9 ± 2.1 | -5.9 ± 0.3 | 34.1 | -8.1 | 43.3 | 2.2 | -9.2 |
| NS6 | GGCUGUCC | -92.0 ± 2.0 | -80.6 ± 3.0 | -11.4 ± 0.6 | 54.3 | -13.4 | 63.2 | 2.0 | -8.9 |
| NS7a | GGCUGUUC | -79.8 ± 1.9 | -71.1 ± 1.7 | -8.7 ± 0.7 | 45.6 | -11.0 | 54.0 | 2.3 | -8.4 |
| NS7b | GGUUCUGC | -76.9 ± 2.1 | -68.5 ± 1.9 | -8.4 ± 0.8 | 44.9 | -11.0 | 54.0 | 2.6 | -9.1 |
| NS8a | AGCUGUCU | -72.6 ± 2.1 | -64.8 ± 1.9 | -7.8 ± 0.9 | 42.4 | -9.9 | 50.8 | 2.1 | -8.4 |
| NS8b | AGUCUGCU | -61.6 ± 0.6 | -53.9 ± 0.6 | -7.7 ± 0.3 | 42.6 | -9.9 | 50.8 | 2.2 | -8.2 |
| NS9a | GGCAGUUC | -75.2 ± 2.3 | -66.5 ± 2.0 | -8.7 ± 0.9 | 46.2 | -11.0 | 54.0 | 2.3 | -7.8 |
| NS9b | GGUUCAGC | -74.9 ± 2.2 | -66.4 ± 2.0 | -8.5 ± 0.9 | 45.1 | -11.0 | 54.0 | 2.5 | -8.9 |
| NS10a | CGCUGUCG | -78.4 ± 2.4 | -68.6 ± 2.1 | -9.8 ± 1.0 | 49.8 | -11.5 | 56.2 | 1.7 | -6.4 |
| NS10b | CGUCUGCG | -75.4 ± 2.4 | -65.7 ± 2.1 | -9.7 ± 1.0 | 50.3 | -11.5 | 56.2 | 1.8 | -5.9 |
| NS11a | UGCUGUCA | -66.2 ± 1.8 | -58.4 ± 1.6 | -7.8 ± 0.7 | 43.0 | -9.8 | 49.6 | 2.0 | -6.6 |
| NS11b | UGUCUGCA | -65.3 ± 0.9 | -57.5 ± 0.8 | -7.8 ± 0.4 | 42.9 | -9.8 | 49.6 | 2.0 | -6.7 |
| NS12a | UAUGAGGA | -71.4 ± 1.4 | -65.4 ± 1.3 | -6.0 ± 0.6 | 34.7 | -8.0 | 41.4 | 2.0 | -6.7 |
| NS12b | UAGAUGGA | -62.7 ± 0.8 | -56.6 ± 0.7 | -6.1 ± 0.3 | 35.2 | -8.0 | 41.4 | 1.9 | -6.2 |
| NS13a | GUGCCGAG | -81.3 ± 1.3 | -70.5 ± 1.1 | -10.8 ± 0.5 | 54.3 | -12.4 | 59.3 | 1.6 | -5.0 |
| NS13b | GCCGAGUG | -71.6 ± 2.1 | -61.2 ± 1.8 | -10.4 ± 0.8 | 54.1 | -12.4 | 59.3 | 2.0 | -5.2 |
| NS14a | CGCUGUAG | -72.2 ± 0.9 | -64.0 ± 0.8 | -8.2 ± 0.4 | 44.1 | -10.2 | 51.7 | 2.0 | -7.6 |
| NS14b | CGUGCUAG | -72.7 ± 1.9 | -64.2 ± 1.7 | -8.5 ± 0.8 | 45.8 | -10.2 | 51.7 | 1.7 | -5.9 |
| NS15a | AUUGGAUACAAA | -105.8 ± 1.4 | -97.4 ± 1.3 | -8.4 ± 0.5 | 42.3 | -10.5 | 48.4 | 2.1 | -6.1 |
| NS15b | AUACAUUGGAAA | -107.5 ± 1.2 | -99.4 ± 1.1 | -8.1 ± 0.5 | 41.6 | -10.5 | 48.4 | 2.4 | -6.8 |
| NS16 | GGCUCAAUUGAC | -118.5 ± 1.5 | -102.8 ± 1.2 | -15.7 ± 0.5 | 56.3 | -16.2 | 61.7 | 0.5 | -5.4 |
| S1 | UACAUGUA | -62.7 ± 2.9 | -59.1 ± 2.8 | -3.6 ± 0.3 | 28.2 | -5.6 | 35.9 | 2.0 | -7.7 |
| S2 | GGUAUACC | -71.9 ± 3.9 | -65.7 ± 3.6 | -6.2 ± 0.5 | 39.2 | -9.1 | 51.8 | 2.9 | -12.6 |
| S3 | GGUUAACC | -73.4 ± 2.8 | -67.7 ± 2.6 | -5.7 ±0.4 | 36.5 | -8.7 | 50.3 | 3.0 | -13.8 |
| S4 | CGAAUUCG | -70.3 ± 2.9 | -65.8 ± 2.7 | -4.5 ± 0.4 | 31.4 | -7.0 | 38.5 | 2.5 | -7.1 |
| S5 | GCUUAAGC | -70.4 ± 3.2 | -64.3 ±2.9 | -6.1 ± 0.4 | 38.6 | -8.4 | 48.1 | 2.3 | -9.5 |

| | | | | | | | | | |
|---|---|---|---|---|---|---|---|---|---|
| S6 | CGUAUACG | -73.8 ± 3.5 | -68.3 ± 3.3 | -5.5 ± 0.5 | 35.7 | -7.3 | 43.9 | 1.8 | -8.2 |
| S7 | CGCAUGCG | -79.3 ±3.7 | -69.7 ± 3.2 | -9.6 ± 0.6 | 52.7 | -10.6 | 55.5 | 1.0 | -2.8 |
| S8 | CGGUACCG | -73.4 ±6.2 | -64.4 ± 5.5 | -9.0 ± 1.0 | 51.0 | -11.7 | 62.3 | 2.7 | -11.3 |
| S9a | CAAGCUUG | -78.3 ± 2.4 | -73.0 ± 2.3 | -5.3 ± 1.0 | 35.8 | -7.4 | 45.3 | 2.1 | -9.5 |
| S9b | CUUGCAAG | -77.5 ± 2.6 | -71.5 ± 2.4 | -6.0 ± 1.1 | 38.3 | -7.4 | 45.3 | 1.4 | -7.0 |
| S10a | GAACGUUC | -73.9 ± 1.2 | -68.2 ± 1.1 | -5.7 ± 0.5 | 36.8 | -7.8 | 45.4 | 2.1 | -8.6 |
| S10b | GUUCGAAC | -83.1 ± 3.4 | -77.9 ± 3.2 | -5.2 ± 1.3 | 35.4 | -7.8 | 45.4 | 2.6 | -10.0 |
| S11a | GAUCCGGAUC | -103.9 ± 1.7 | -91.6 ± 1.5 | -12.3 ± 0.7 | 58.4 | -13.8 | 60.6 | 1.5 | -2.2 |
| S11b | GGAUCGAUCC | -100.1 ± 1.8 | -88.7 ± 1.6 | -11.4 ± 0.7 | 56.0 | -13.8 | 60.6 | 2.4 | -4.6 |
| S12a | AUGAGCUCAU | -80.7 ± 2.3 | -72.1 ± 2.0 | -8.6 ± 0.3 | 48.8 | -11.3 | 56.7 | 2.7 | -7.9 |
| S12b | AUCAGCUGAU | -83.3 ± 1.3 | -75.1 ± 1.1 | -8.2 ± 0.5 | 46.7 | -11.3 | 56.7 | 3.1 | -10.0 |
| S13 | UUACGCGUAA | -78.9 ±4.7 | -71.7 ± 4.2 | -7.2 ±0.6 | 42.2 | -10.5 | 54.6 | 3.3 | -12.4 |
| S14 | AUCGCUAGCGAU | -105.3 ± 4.7 | -92.3 ± 4.8 | -13.0 ± 0.8 | 60.0 | -16.0 | 65.5 | 3.0 | -5.5 |
| S15a | CAUAGGCCUAUG | -130.9 ± 2.1 | -116.5 ± 1.9 | -14.4 ± 0.7 | 59.1 | -16.5 | 68.2 | 2.1 | -9.1 |
| S15b | CUAUGGCCAUAG | -131.7 ± 2.2 | -117.2 ± 2.2 | -14.5 ± 0.7 | 59.4 | -16.5 | 68.2 | 2.0 | -8.8 |

[a]RNA duplex consists of a denoted RNA strand (5' → 3') and complementary RNA strand. The pair of RNA oligonucleotides with identical nearest neighbors are shown by the numbers a and b. [b]All experiments were performed in a buffer containing 100 mM NaCl, 10 mM $Na_2HPO_4$ (pH 7.0), and 1 mM $Na_2$EDTA. [c]The values in the absence of cosolute were calculated from the parameters of 121 mM $Na^+$ reported by Weber *et al.*(4) (Table S3) [d]Melting temperatures were calculated for a total strand concentration of 100 μM.

**Table S3**. NN parameters for RNA duplex formation in the absence of cosolute in 100 mM NaCl at 37 °C[a]

| Sequence | $\Delta H°_{NN}$ (kcal mol$^{-1}$) | $\Delta S°_{NN}$ (cal mol$^{-1}$ K$^{-1}$) | $\Delta G°_{37,\ NN,\ no\ cosolute}$ (kcal mol$^{-1}$) |
|---|---|---|---|
| r(AA/UU) | -10.7 | -32.2 | -0.70 |
| r(AU/UA) | -7.6 | -22.8 | -0.52 |
| r(UA/AU) | -11.5 | -32.9 | -1.30 |
| r(CA/GU) | -11.7 | -32.0 | -1.78 |
| r(GU/CA) | -11.2 | -29.2 | -2.14 |
| r(CU/GA) | -9.6 | -25.1 | -1.80 |
| r(GA/CU) | -14.8 | -40.6 | -2.22 |
| r(CG/GC) | -10.9 | -28.1 | -2.18 |
| r(GC/CG) | -15.1 | -38.0 | -3.30 |
| r(GG/CC) | -12.9 | -31.6 | -3.10 |
| initiation | 3.6 | -1.5 | 4.09 |
| per terminal AU | 3.7 | 10.5 | 0.45 |
| self-complementary[b] | 0 | -1.4 | 0.43 |
| non-self-complementary | 0 | 0 | 0 |

[a]Parameters are collected from the report by Weber et al.(4) [b]Symmetry factors for self-complementary sequences are independent of the solution conditions.

**Table S4**. Stability contribution of 40 wt% PEG200 ($\Delta\Delta G°_{37,\ [40\ wt\%\ PEG200]} = \Delta G°_{37,\ [40\ wt\%\ PEG200,\ 100\ mM\ NaCl]} - \Delta G°_{37,\ [no\ cosolute,\ 100\ mM\ NaCl]}$) on the individual nearest-neighbor base pairs for RNA duplex formation at 100 mM NaCl

| Sequence | $\Delta\Delta G°_{37,\ [40\ wt\%\ PEG200]}$[a] (kcal mol$^{-1}$) |
|---|---|
| r(AA/UU) | 0.13 |
| r(AU/UA) | -0.03 |
| r(UA/AU) | -0.03 |
| r(CA/GU) | -0.36 |
| r(GU/CA) | 0.34 |
| r(CU/GA) | 0.03 |
| r(GA/CU) | -0.02 |
| r(CG/GC) | 0.02 |
| r(GC/CG) | 0.23 |
| r(GG/CC) | 0.21 |
| initiation | 1.41 |
| per terminal AU | 0.40 |

[a]$\Delta G°_{37,\ [40\ wt\%\ PEG200,\ 100\ mM\ NaCl]}$ were taken from Table 1 and $\Delta G°_{37,\ [no\ cosolute,\ 100\ mM\ NaCl]}$ were collected from Table S3.

**Table S5.** Measured and predicted thermodynamic parameters for RNA duplex formation with 40 wt% PEG200 and 100 mM NaCl in 10 mM phosphate buffer (pH 7.0)

| | | Measured[b] | | | | Predicted | | | |
|---|---|---|---|---|---|---|---|---|---|
| No. | Sequence[a] | $\Delta H°$ (kcal mol$^{-1}$) | $\Delta S°$ (cal mol$^{-1}$ K$^{-1}$) | $\Delta G°_{37}$ (kcal mol$^{-1}$) | $T_m$[c] (°C) | $\Delta H°$ (kcal mol$^{-1}$) | $\Delta S°$ (cal mol$^{-1}$K$^{-1}$) | $\Delta G°_{37}$ (kcal mol$^{-1}$) | $T_m$[c] (°C) |
| NS1 | CAUGCC | -56.5 | -164.1 | -5.6 | 31.0 | -58.3 | -170.9 | -5.3 | 30.6 |
| NS2 | CUAGGC | -57.5 | -165.7 | -6.1 | 34.3 | -57.5 | -168.2 | -5.3 | 30.7 |
| NS3 | CAGCGG | -65.6 | -190.9 | -6.4 | 36.4 | -58.4 | -167.2 | -6.5 | 37.1 |
| NS4 | CUACGC | -62.8 | -187.3 | -4.7 | 27.8 | -53.3 | -156.9 | -4.6 | 26.4 |
| NS5 | GAACUCC | -71.8 | -212.5 | -5.9 | 34.1 | -65.5 | -191.8 | -6.0 | 34.6 |
| NS6 | GGCUGUCC | -92.0 | -259.9 | -11.4 | 54.3 | -84.5 | -236.0 | -11.3 | 55.6 |
| NS7a | GGCUGUUC | -79.8 | -229.2 | -8.7 | 45.6 | -79.7 | -228.0 | -9.0 | 46.9 |
| NS7b | GGUUCUGC | -76.9 | -220.9 | -8.4 | 44.9 | -79.7 | -228.0 | -9.0 | 46.9 |
| NS8a | AGCUGUCU | -72.6 | -208.9 | -7.8 | 42.4 | -64.3 | -183.6 | -7.4 | 41.0 |
| NS8b | AGUCUGCU | -61.6 | -173.8 | -7.7 | 42.6 | -64.3 | -183.6 | -7.4 | 41.0 |
| NS9a | GGCAGUUC | -75.2 | -214.4 | -8.7 | 46.2 | -79.7 | -228.0 | -9.0 | 46.9 |
| NS9b | GGUUCAGC | -74.9 | -214.1 | -8.5 | 45.1 | -79.7 | -228.0 | -9.0 | 46.9 |
| NS10a | CGCUGUCG | -78.4 | -221.2 | -9.8 | 49.8 | -77.1 | -216.8 | -9.8 | 51.0 |
| NS10b | CGUCUGCG | -75.4 | -211.8 | -9.7 | 50.3 | -77.1 | -216.8 | -9.8 | 51.0 |
| NS11a | UGCUGUCA | -66.2 | -188.3 | -7.8 | 43.0 | -66.1 | -187.0 | -8.1 | 44.6 |
| NS11b | UGUCUGCA | -65.3 | -185.4 | -7.8 | 42.9 | -66.1 | -187.0 | -8.1 | 44.6 |
| NS12a | UAUGAGGA | -71.4 | -210.9 | -6.0 | 34.7 | -65.1 | -190.7 | -6.0 | 34.3 |
| NS12b | UAGAUGGA | -62.7 | -182.5 | -6.1 | 35.2 | -65.1 | -190.7 | -6.0 | 34.3 |
| NS13a | GUGCCGAG | -81.3 | -227.3 | -10.8 | 54.3 | -80.8 | -226.4 | -10.6 | 53.4 |
| NS13b | GCCGAGUG | -71.6 | -197.3 | -10.4 | 54.1 | -80.8 | -226.4 | -10.6 | 53.4 |
| NS14a | CGCUGUAG | -72.2 | -206.4 | -8.2 | 44.1 | -76.6 | -219.4 | -8.5 | 45.4 |
| NS14b | CGUGCUAG | -72.7 | -207.0 | -8.5 | 45.8 | -76.6 | -219.4 | -8.5 | 45.4 |
| NS15a | AUUGGAUACAAA | -105.8 | -314.0 | -8.4 | 42.3 | -105.1 | -312.6 | -8.2 | 41.8 |
| NS15b | AUACAUUGGAAA | -107.5 | -320.5 | -8.1 | 41.6 | -105.1 | -312.6 | -8.2 | 41.8 |
| NS16 | GGCUCAAUUGAC | -118.5 | -331.5 | -15.7 | 56.3 | -123.6 | -351.8 | -14.5 | 58.3 |
| S1 | UACAUGUA | -62.7 | -190.6 | -3.6 | 28.2 | -60.3 | -183.3 | -3.5 | 26.0 |
| S2 | GGUAUACC | -71.9 | -211.8 | -6.2 | 39.2 | -78.7 | -232.3 | -6.7 | 40.9 |
| S3 | GGUUAACC | -73.4 | -218.3 | -5.7 | 36.5 | -77.5 | -230.8 | -5.9 | 38.0 |
| S4 | CGAAUUCG | -70.3 | -212.2 | -4.5 | 31.4 | -71.1 | -214.5 | -4.6 | 32.3 |
| S5 | GCUUAAGC | -70.4 | -207.3 | -6.1 | 38.6 | -76.5 | -226.6 | -6.2 | 39.2 |
| S6 | CGUAUACG | -73.8 | -220.2 | -5.5 | 35.7 | -71.3 | -213.1 | -5.2 | 35.0 |
| S7 | CGCAUGCG | -79.3 | -224.7 | -9.6 | 52.7 | -79.5 | -226.1 | -9.4 | 52.1 |

| | | | | | | | | | |
|---|---|---|---|---|---|---|---|---|---|
| S8 | CGGUACCG | -73.4 | -207.6 | -9.0 | 51.0 | -79.7 | -227.6 | -9.1 | 51.0 |
| S9a | CAAGCUUG | -78.3 | -235.4 | -5.3 | 35.8 | -75.8 | -224.7 | -6.1 | 38.8 |
| S9b | CUUGCAAG | -77.5 | -230.5 | -6.0 | 38.3 | -75.8 | -224.7 | -6.1 | 38.8 |
| S10a | GAACGUUC | -73.9 | -219.9 | -5.7 | 36.8 | -71.3 | -212.3 | -5.5 | 36.0 |
| S10b | GUUCGAAC | -83.1 | -251.2 | -5.2 | 35.4 | -71.3 | -212.3 | -5.5 | 36.0 |
| S11a | GAUCCGGAUC | -103.9 | -295.3 | -12.3 | 58.4 | -103.1 | -293.5 | -12.1 | 57.5 |
| S11b | GGAUCGAUCC | -100.1 | -286.0 | -11.4 | 56.0 | -103.1 | -293.5 | -12.1 | 57.5 |
| S12a | AUGAGCUCAU | -80.7 | -232.5 | -8.6 | 48.8 | -86.4 | -250.1 | -8.8 | 48.8 |
| S12b | AUCAGCUGAU | -83.3 | -242.1 | -8.2 | 46.7 | -86.4 | -250.1 | -8.8 | 48.8 |
| S13 | UUACGCGUAA | -78.9 | -231.2 | -7.2 | 42.2 | -82.0 | -241.3 | -7.2 | 42.7 |
| S14 | AUCGCUAGCGAU | -105.3 | -297.6 | -13.0 | 60.0 | -109.3 | -309.6 | -13.3 | 60.2 |
| S15a | CAUAGGCCUAUG | -130.9 | -375.6 | -14.4 | 59.1 | -127.8 | -365.3 | -14.5 | 60.0 |
| S15b | CUAUGGCCAUAG | -131.7 | -377.9 | -14.5 | 59.4 | -127.8 | -365.3 | -14.5 | 60.0 |

[a]RNA duplex consists of denoted RNA strand (5′ → 3′) and complementary RNA strand. [b]Errors associated with the measured values were mentioned in the Table S2. [c]Melting temperatures were calculated for total strand concentration of 100 μM.

**Table S6**. Parameters for RNA-PEG excluded volume calculations

| Sequence | $R$ [a] (Å) | $L$ [b] (Å) | $P$ [c] | $C(p)$ [d] | $k$ [e] (Å) | $V/N$ [f] (L $mol^{-1}$) |
|---|---|---|---|---|---|---|
| 6-bp duplex | 10.0 | 20.4 | 2.04 | 1.20 | 8.88 | 0.76 |
| 6-nt strand | 5.0 | 20.4 | 4.08 | 1.76 | 6.09 | 0.52 |
| 12-bp duplex | 10.0 | 40.8 | 4.08 | 1.76 | 12.17 | 1.04 |
| 12-nt strand | 5.0 | 40.8 | 8.16 | 2.37 | 9.01 | 0.77 |
| 24-bp duplex | 10.0 | 81.6 | 8.16 | 2.37 | 18.03 | 1.54 |
| 24-nt strand | 5.0 | 81.6 | 16.32 | 3.03 | 14.12 | 1.20 |
| 36-bp duplex | 10.0 | 122.4 | 12.24 | 2.75 | 23.29 | 1.99 |
| 36-nt strand | 5.0 | 122.4 | 24.48 | 3.42 | 18.75 | 1.60 |
| 34-nt hairpin | 10.0 | 57.8 | 5.78 | 2.06 | 14.71 | 1.25 |
| 34-nt strand | 5.0 | 115.6 | 23.12 | 3.36 | 18.01 | 1.54 |

[a] Radius of equivalent cylinder. [b]Length of equivalent cylinder. [a, b] Values are collected from the report of Knowles et al. (5) following both DNA and RNA oligomeric duplexes has almost same radius and length.(6) [c]$p = L/R$. [d]$C(p) = (1+p^2)^{3/2}/3p - p^2/3 + 2/3p - (1+p^2)^{1/2}/p + \ln [p+(1+p^2)^{1/2}]$. [e]$k = \pi pR/6C(p)$. [f]Obtained by using equation 10 and Kuhn length ($l$) of PEG was taken as 11.9 Å.(5)

**Table S7**. Excluded volume and energy contribution of excluded volume for different lengths of RNA duplexes with PEG

| Sequence | $\Delta V/N$ [a] (L mol$^{-1}$) | $\Delta G°_{37,\text{ ev, dx}}/C•N$ [b] (kcal mol$^{-2}$ kg) |
|---|---|---|
| 6-bp duplex | -0.28 | -0.17 |
| 12-bp duplex | -0.50 | -0.31 |
| 24-bp duplex | -0.86 | -0.52 |
| 36-bp duplex | -1.21 | -0.74 |

[a]Calculated from the values in Table S6 using equation 9. [b]Calculated by using equation 8 and taking density of water 0.99 kg L$^{-1}$ at 37 °C.(5)

**Table S8**. Thermodynamic parameters of GAUUACGCCUG in different cosolute solutions and the water activity values of the solutions

| Solution | $\Delta H°$ (kcal mol$^{-1}$) | $T\Delta S°$ (cal mol$^{-1}$ K$^{-1}$) | $\Delta G°_{37}$ (kcal mol$^{-1}$) | $\Delta G°_{37,\text{ ev, dup}}$[a] (kcal mol$^{-1}$) | $\Delta G°_{37,\text{ wa, dup}}$[b] (kcal mol$^{-1}$) | Water activity[c] |
|---|---|---|---|---|---|---|
| In 100 mM NaCl | | | | | | |
| No cosolute | -97.8 ± 1.6 | -83.7 ± 1.5 | -14.1 ± 0.1 | | | 0.996 |
| 10 wt% EG | -97.9 ± 4.0 | -84.3 ± 3.8 | -13.6 ± 0.3 | -0.45 | 0.95 | 0.970 |
| 15 wt% EG | -101.1 ± 1.0 | -87.7 ± 0.9 | -13.4 ± 0.1 | -0.68 | 1.38 | 0.959 |
| 5 wt% Gly | -95.4 ± 2.6 | -81.5 ± 2.3 | -13.9 ± 0.3 | -0.15 | 0.35 | 0.987 |
| 10 wt% Gly | -98.4 ± 1.6 | -84.4 ± 1.5 | -13.6 ± 0.1 | -0.30 | 0.80 | 0.975 |
| 15 wt% Gly | -98.6 ± 1.0 | -85.2 ± 0.8 | -13.4 ± 0.1 | -0.45 | 1.15 | 0.965 |
| 20 wt% Gly | -97.3 ± 1.7 | -84.2 ± 1.6 | -13.1 ± 0.1 | -0.61 | 1.61 | 0.951 |
| 5 wt% 1,3 PDO | -100.1 ± 1.2 | -86.2 ± 1.1 | -13.9 ± 0.1 | -0.18 | 0.38 | 0.985 |
| 10 wt% 1,3 PDO | -100.4 ± 3.1 | -86.7 ± 2.9 | -13.7 ± 0.3 | -0.37 | 0.77 | 0.971 |
| 20 wt% 1,3 PDO | -103.5 ± 0.5 | -90.0 ± 0.5 | -13.5 ± 0.1 | -0.73 | 1.33 | 0.947 |
| 40 wt% 1,3 PDO | -105.8 ± 2.2 | -92.9 ± 2.1 | -12.9 ± 0.1 | -1.47 | 2.67 | 0.907 |
| 10 wt% 2-ME | -100.8 ± 1.6 | -87.7 ± 1.6 | -13.1 ± 0.1 | -0.37 | 1.37 | 0.975 |
| 40 wt% 1,2-DME | -94.7 ± 5.5 | -81.5 ± 5.1 | -13.2 ± 0.3 | -1.24 | 2.14 | 0.965 |
| 10 wt% PEG200 | -102.2 ± 1.8 | -88.3 ± 1.6 | -13.9 ± 0.2 | -0.56 | 0.76 | 0.985 |
| 20 wt% PEG200 | -100.7 ± 1.3 | -87.3 ± 1.2 | -13.4 ± 0.1 | -1.12 | 1.82 | 0.971 |
| 30 wt% PEG200 | -101.4 ± 1.7 | -88.4 ± 1.6 | -13.0 ± 0.2 | -1.67 | 2.77 | 0.956 |
| 40 wt% PEG200 | -96.2 ± 1.9 | -84.2 ± 1.8 | -12.0 ± 0.2 | -2.23 | 4.33 | 0.947 |
| 50 wt% PEG200 | -90.6 ± 1.0 | -79.6 ± 1.0 | -11.0 ± 0.1 | -2.79 | 5.89 | 0.931 |
| 20 wt% PEG400 | -96.0 ± 5.4 | -82.5 ± 5.1 | -13.5 ± 0.3 | -1.12 | 1.72 | 0.980 |
| 20 wt% PEG600 | -103.5 ± 2.9 | -88.9 ± 2.7 | -14.6 ± 0.2 | -1.21 | 0.71 | 0.983 |
| In 1 M NaCl | | | | | | |
| No cosolute | -98.4 ± 1.2 | -81.9 ± 1.2 | -16.5 ± 0.1 | | | 0.967 |
| 20 wt% EG | -99.8 ± 1.9 | -84.8 ± 1.8 | -15.0 ± 0.1 | -0.90 | 2.40 | 0.907 |
| 15 wt% 1,3 PDO | -103.8 ± 2.1 | -88.0 ± 2.0 | -15.8 ± 0.2 | -0.55 | 1.25 | 0.933 |
| 15 wt% 2-ME | -99.5 ± 0.6 | -84.9 ± 0.5 | -14.6 ± 0.1 | -0.55 | 2.45 | 0.927 |
| 20 wt% 1,2-DME | -99.0 ± 2.7 | -83.8 ± 2.7 | -15.2 ± 0.2 | -0.62 | 1.92 | 0.934 |
| 10 wt% PEG200 | -98.5 ± 1.8 | -82.4 ± 1.6 | -16.1 ± 0.1 | -0.56 | 0.96 | 0.953 |
| 20 wt% PEG200 | -100.5 ± 2.4 | -85.1 ± 2.2 | -15.4 ± 0.2 | -1.12 | 2.22 | 0.939 |
| 40 wt% PEG200 | -92.3 ± 2.3 | -78.9 ± 2.7 | -13.4 ± 0.7 | -2.23 | 5.33 | 0.911 |

[a]Calculated by using equation 11. Excluded volume for Gly, 1,3 PDO, 2-ME and 1,2-DME were considered to be same as EG due to similar molecular weights. [b]$\Delta G°_{37,\text{ wa, dup}} = \Delta G°_{37} - \Delta G°_{37,\text{ no cosolute}} - \Delta G°_{37,\text{ ev, dup}}$. [c]Water activities were calculated at 37 °C from corresponding osmolality values of respective solutions calculated as described in Methods section. Error limit was ± 0.002.

**Table S9.** Thermodynamic parameters for RNA duplexes measured in the presence of NaCl and KCl with 40 wt% PEG200

| | | NaCl[b] | | | | KCl[c] | | | |
|---|---|---|---|---|---|---|---|---|---|
| No. | Sequence[a] | $\Delta H°$ (kcal mol$^{-1}$) | $T\Delta S°$ (cal mol$^{-1}$ K$^{-1}$) | $\Delta G°_{37}$ (kcal mol$^{-1}$) | $T_m$[d] (°C) | $\Delta H°$ (kcal mol$^{-1}$) | $T\Delta S°$ (cal mol$^{-1}$K$^{-1}$) | $\Delta G°_{37}$ (kcal mol$^{-1}$) | $T_m$[d] (°C) |
| NS3 | CAGCGG | -65.6 ± 3.0 | -59.2 ± 2.7 | -6.4 ± 0.4 | 36.4 | -55.2 ± 2.7 | -49.1 ± 2.4 | -6.1 ± 0.4 | 34.3 |
| NS5 | GAACUCC | -71.8 ± 2.3 | -65.9 ± 2.1 | -5.9 ± 0.3 | 34.1 | -63.2 ± 1.9 | -57.8 ± 1.8 | -5.4 ± 0.3 | 31.2 |
| NS10a | CGCUGUCG | -78.4 ± 2.4 | -68.6 ± 2.1 | -9.8 ± 1.0 | 49.8 | -71.9 ± 0.7 | -63.1 ± 0.6 | -8.8 ± 0.1 | 47.1 |
| NS11a | UGCUGUCA | -66.2 ± 1.8 | -58.4 ± 1.6 | -7.8 ± 0.7 | 43.0 | -62.6 ± 1.6 | -55.6 ± 1.4 | -7.0 ± 0.2 | 39.6 |
| NS13a | GUGCCGAG | -81.3 ± 1.3 | -70.5 ± 1.1 | -10.8 ± 0.5 | 54.3 | -72.5 ± 3.7 | -62.8 ± 3.2 | -9.7 ± 0.6 | 51.1 |
| NS13b | GCCGAGUG | -71.6 ± 2.1 | -61.2 ± 1.8 | -10.4 ± 0.8 | 54.1 | -71.8 ± 5.4 | -62.0 ± 4.7 | -9.8 ± 0.9 | 51.6 |
| S2 | GGUAUACC | -71.9 ± 3.9 | -65.7 ± 3.6 | -6.2 ± 0.5 | 39.2 | -72.3 ± 2.0 | -66.8 ± 1.8 | -5.5 ± 0.3 | 36.1 |
| S3 | GGUUAACC | -73.4 ± 2.8 | -67.7 ± 2.6 | -5.7 ± 0.4 | 36.5 | -66.7 ± 3.3 | -61.7 ± 3.1 | -5.0 ± 0.5 | 33.6 |
| S5 | GCUUAAGC | -70.4 ± 3.2 | -64.3 ± 2.9 | -6.1 ± 0.4 | 38.6 | -64.2 ± 1.1 | -59.1 ± 1.0 | -5.1 ± 0.1 | 34.1 |
| S7 | CGCAUGCG | -79.3 ± 3.7 | -69.7 ± 3.2 | -9.6 ± 0.6 | 52.7 | -75.1 ± 2.9 | -66.6 ± 2.6 | -8.5 ± 0.5 | 48.6 |
| S8 | CGGUACCG | -73.4 ± 6.2 | -64.4 ± 5.5 | -9.0 ± 1.0 | 52.7 | -69.9 ± 3.5 | -61.0 ± 3.1 | -8.6 ± 0.6 | 50.4 |
| S11a | GAUCCGGAUC | -103.9 ± 1.7 | -91.6 ± 1.5 | -12.3 ± 0.7 | 58.4 | -105.3 ± 4.1 | -93.9 ± 3.7 | -11.4 ± 0.6 | 55.0 |
| S12a | AUGAGCUCAU | -80.7 ± 2.3 | -72.1 ± 2.0 | -8.6 ± 0.3 | 48.8 | -79.6 ± 3.5 | -72.2 ± 3.2 | -7.4 ± 0.5 | 44.1 |
| S12b | AUCAGCUGAU | -83.3 ± 1.3 | -75.1 ± 1.1 | -8.2 ± 0.5 | 46.7 | -79.8 ± 2.7 | -72.8 ± 2.4 | -7.0 ± 0.4 | 42.1 |
| S13 | UUACGCGUAA | -78.9 ± 4.7 | -71.7 ± 4.2 | -7.2 ± 0.6 | 42.2 | -77.7 ± 1.7 | -71.5 ± 1.5 | -6.2 ± 0.2 | 38.8 |
| S14 | AUCGCUAGCGAU | -105.3 ± 4.7 | -92.3 ± 4.8 | -13.0 ± 0.8 | 60.0 | -100.7 ± 1.9 | -89.4 ± 1.6 | -11.3 ± 0.3 | 55.4 |

[a]RNA duplex consists of denoted RNA strand (5' → 3') and complementary RNA strand. [b]Experiments were performed in a buffer containing 100 mM NaCl, 10 mM $Na_2HPO_4$, and 1 mM $Na_2EDTA$ in 40 wt% PEG200 at pH 7.0. Values were collected from Table S2. [c]Experiments were performed in a buffer containing 100 mM KCl, 10 mM $K_2HPO_4$, and 1 mM $K_2EDTA$ in 40 wt% PEG200 at pH 7.0. [d]Melting temperatures were calculated for total strand concentration of 100 µM.

**Table S10.** Dielectric constants ($\varepsilon_r$) of the NaCl and KCl solutions in the presence and absence of cosolutes at 37 °C

| Solutions | $\varepsilon_r$ |
|---|---|
| In 100 mM NaCl with 10 mM $Na_2HPO_4$, and 1 mM $Na_2EDTA$ | |
| absence of cosolute | 76.8 ± 0.6 |
| 40 wt% PEG200 | 39.4 ± 0.4 |
| 40 wt% EG | 62.0 ± 0.5 |
| In 100 mM KCl with 10 mM $K_2HPO_4$, and 1 mM $K_2EDTA$ | |
| absence of cosolute | 74.5 ± 0.6 |
| 40 wt% PEG200 | 42.5 ± 0.4 |
| 40 wt% EG | 63.2 ± 0.5 |

**Table S11.** Thermodynamic parameters for RNA duplexes measured in the presence of NaCl and KCl without any cosolute

| | | NaCl[b] | | | | KCl[c] | | | |
|---|---|---|---|---|---|---|---|---|---|
| No. | Sequence[a] | $\Delta H°$ (kcal mol$^{-1}$) | $T\Delta S°$ (cal mol$^{-1}$ K$^{-1}$) | $\Delta G°_{37}$ (kcal mol$^{-1}$) | $T_m$[d] (°C) | $\Delta H°$ (kcal mol$^{-1}$) | $T\Delta S°$ (cal mol$^{-1}$K$^{-1}$) | $\Delta G°_{37}$ (kcal mol$^{-1}$) | $T_m$[d] (°C) |
| NS1 | CAUGCC | -62.0 ± 3.2 | -55.0 ± 2.9 | -7.0 ± 0.5 | 39.2 | -57.6 ± 5.4 | -50.7 ± 4.8 | -6.9 ± 0.8 | 38.5 |
| NS2 | CUAGGC | -54.7 ± 5.5 | -47.1 ± 4.9 | -7.6 ± 0.9 | 42.5 | -69.4 ± 7.3 | -62.0 ± 6.6 | -7.4 ± 1.0 | 40.7 |
| NS5 | GAACUCC | -67.3 ± 3.5 | -59.4 ± 3.2 | -7.9 ± 0.5 | 43.1 | -71.1 ± 4.1 | -63.5 ± 3.7 | -7.6 ± 0.6 | 41.6 |
| NS11a | UGCUGUCA | -71.8 ± 1.7 | -61.6 ± 1.5 | -10.2 ± 0.3 | 54.1 | -69.4 ± 1.8 | -59.5 ± 1.5 | -9.9 ± 0.3 | 53.1 |
| S6 | CGUAUACG | -74.2 ± 0.4 | -67.0 ± 0.3 | -7.2 ± 0.1 | 43.3 | -77.3 ± 3.0 | -70.4 ± 2.8 | -6.9 ± 0.4 | 41.6 |
| S8 | CGGUACCG | -85.3 ± 1.9 | -73.8 ± 1.6 | -11.5 ± 0.3 | 59.9 | -82.0 ± 2.6 | -70.6 ± 2.3 | -11.4 ± 0.4 | 60.0 |
| S12a | AUGAGCUCAU | -83.2 ± 8.0 | -71.6 ± 6.9 | -11.6 ± 1.3 | 60.9 | -77.0 ± 3.8 | -66.0 ± 3.3 | -11.0 ± 0.7 | 59.8 |
| S13 | UUACGCGUAA | -85.2 ± 3.1 | -75.1 ± 2.8 | -10.1 ± 0.5 | 53.6 | -84.8 ± 3.7 | -75.1 ± 3.3 | -9.7 ± 0.6 | 52.1 |

[a]RNA duplex consists of denoted RNA strand (5′ → 3′) and complementary RNA strand. [b]Experiments were performed in a buffer containing 100 mM NaCl, 10 mM $Na_2HPO_4$, and 1 mM $Na_2EDTA$ at pH 7.0. [c]Experiments were performed in a buffer containing 100 mM KCl, 10 mM $K_2HPO_4$, and 1 mM $K_2EDTA$ at pH 7.0. [d]Melting temperatures were calculated for total strand concentration of 100 µM.

**Table S12.** Thermodynamic parameters for RNA duplexes measured in the presence of NaCl and KCl with 40 wt% EG

| | | NaCl[b] | | | | KCl[c] | | | |
|---|---|---|---|---|---|---|---|---|---|
| No. | Sequence[a] | Δ*H*° (kcal mol$^{-1}$) | *T*Δ*S*° (cal mol$^{-1}$ K$^{-1}$) | Δ*G*°$_{37}$ (kcal mol$^{-1}$) | $T_m$[d] (°C) | Δ*H*° (kcal mol$^{-1}$) | *T*Δ*S*° (cal mol$^{-1}$K$^{-1}$) | Δ*G*°$_{37}$ (kcal mol$^{-1}$) | $T_m$[d] (°C) |
| NS2 | CUAGGC | -58.4 ± 6.1 | -52.3 ± 5.8 | -6.1 ± 0.8 | 33.8 | -55.5 ± 3.2 | -49.7 ± 2.9 | -5.8 ± 0.4 | 33.2 |
| NS5 | GAACUCC | -65.3 ± 1.4 | -59.2 ± 1.2 | -6.1 ± 0.2 | 35.2 | -65.5 ± 5.6 | -59.7 ± 5.2 | -5.8 ± 0.7 | 33.9 |
| S2 | GGUAUACC | -69.3 ± 4.7 | -63.5 ± 4.3 | -5.8 ± 0.7 | 36.0 | -72.0 ± 3.2 | -66.4 ± 3.0 | -5.6 ± 0.4 | 36.5 |
| S3 | GGUUAACC | -71.4 ± 2.3 | -66.1 ± 2.1 | -5.3 ± 0.3 | 35.1 | -67.2 ± 2.2 | -62.0 ± 2.0 | -5.2 ± 0.3 | 34.7 |
| S5 | GCUUAAGC | -68.3 ± 2.7 | -62.6 ± 2.5 | -5.7 ± 0.4 | 36.5 | -64.1 ± 1.2 | -58.8 ± 1.1 | -5.3 ± 0.2 | 35.0 |
| S8 | CGGUACCG | -77.2 ± 4.2 | -68.3 ± 3.7 | -8.9 ± 0.7 | 50.1 | -74.8 ± 0.9 | -66.0 ± 0.8 | -8.8 ± 0.1 | 50.2 |
| S12a | AUGAGCUCAU | -80.4 ± 2.0 | -71.9 ± 1.8 | -8.5 ± 0.3 | 48.1 | -75.1 ± 6.4 | -67.1 ± 5.7 | -8.0 ± 0.9 | 46.8 |
| S13 | UUACGCGUAA | -88.0 ± 2.8 | -81.3 ± 2.6 | -6.7 ± 0.4 | 40.8 | -89.4 ± 5.8 | -83.0 ± 5.4 | -6.4 ± 0.7 | 39.8 |

[a]RNA duplex consists of denoted RNA strand (5′ → 3′) and complementary RNA strand. [b]Experiments were performed in a buffer containing 100 mM NaCl, 10 mM $Na_2HPO_4$, and 1 mM $Na_2$EDTA in 40 wt% EG at pH 7.0. [c]Experiments were performed in a buffer containing 100 mM KCl, 10 mM $K_2HPO_4$, and 1 mM $K_2$EDTA in 40 wt% EG at pH 7.0. [d]Melting temperatures were calculated for total strand concentration of 100 µM.

**Table S13.** Measured and calculated $\Delta G°_{37}$ for RNA duplexes under the molecular crowding environment of 40 wt% PEG200 with 100 mM KCl

| No. | Sequence[a] | Measured $\Delta G°_{37}$ [b] (kcal mol$^{-1}$) | Calculated $\Delta G°_{37}$ [c] (kcal mol$^{-1}$) |
|---|---|---|---|
| NS3 | CAGCGG | -6.1 | -5.6 |
| NS5 | GAACUCC | -5.4 | -5.1 |
| NS10a | CGCUGUCG | -8.8 | -8.7 |
| NS11a | UGCUGUCA | -7.0 | -7.1 |
| NS13a | GUGCCGAG | -9.7 | -9.4 |
| NS13b | GCCGAGUG | -9.8 | -9.4 |
| S2 | GGUAUACC | -5.5 | -5.8 |
| S3 | GGUUAACC | -5.0 | -5.0 |
| S5 | GCUUAAGC | -5.1 | -5.3 |
| S7 | CGCAUGCG | -8.5 | -8.3 |
| S8 | CGGUACCG | -8.6 | -8.0 |
| S11a | GAUCCGGAUC | -11.4 | -10.8 |
| S12a | AUGAGCUCAU | -7.4 | -7.7 |
| S12b | AUCAGCUGAU | -7.0 | -7.7 |
| S13 | UUACGCGUAA | -6.2 | -6.4 |
| S14 | AUCGCUAGCGAU | -11.3 | -11.9 |

[a]RNA duplex consists of denoted RNA strand (5′ → 3′) and complementary RNA strand. [b]Errors associated with the measured values were mentioned in the Table S9. [c]Calculated using equation 15 and the parameters of Table 1.

**Table S14.** Measured and predicted $\Delta G°_{37}$ of RNA duplexes in different cosolutes and NaCl concentrations

| Solution/Sequences[a] | Measured $\Delta G°_{37}$ (kcal mol$^{-1}$) | Predicted $\Delta G°_{37}$ (kcal mol$^{-1}$) |
|---|---|---|
| GGCUCAAUUGAC in 100 mM NaCl | | |
| 10 wt% PEG200 | -15.1 ± 0.8 | -15.4 |
| 20 wt% PEG200 | -14.8 ± 0.6 | -14.7 |
| 30 wt% PEG200 | -14.0 ± 0.7 | -14.0 |
| 40 wt% PEG200 | -13.8 ± 0.6 | -13.7 |
| In 20 wt% EG at 100 mM NaCl | | |
| GGAUCGAUCC | -12.7 ± 0.7 | -13.1 |
| AUCAGCUGAU | -9.9 ± 0.6 | -9.9 |
| GGCUCAAUUGAC | -14.4 ± 0.6 | -15.0 |
| In 20 wt% 1,3 PDO at 100 mM NaCl | | |
| GAUCCGGAUC | -14.5 ± 0.7 | -12.9 |
| GGCUCAAUUGAC | -14.7 ± 0.6 | -14.7 |
| In 20 wt% PEG2000 at 100 mM NaCl[b] | | |
| GGCUCAAUUGAC | -16.9 ± 0.8 | -16.3 |
| GAUUACGCCUG | -15.5 ± 0.5 | -15.4 |
| In 20 wt% PEG8000 at 100 mM NaCl[c] | | |
| GGCUCAAUUGAC | -16.7 ± 0.4 | -16.5 |
| GGCUCAAUUGAC in 1 M NaCl | | |
| 10 wt% PEG200 | -18.9 ± 0.8 | -17.9 |
| 20 wt% PEG200 | -18.3 ± 0.6 | -17.2 |
| In 20 vol% PEG200 at 1 M NaCl[d] | | |
| UCAUGA[e] | -3.0 ± 0.1 | -3.8 |
| ACUGCG[e] | -6.7 ± 0.0 | -7.2 |
| AUGGAC[e] | -5.9 ± 0.1 | -6.1 |
| GCGAUA[e] | -5.3 ± 0.2 | -5.6 |
| GCUAUG[e] | -5.2 ± 0.2 | -5.2 |
| ACCGGU[e] | -7.1 ± 0.1 | -6.0 |
| AGCGCU[e] | -6.8 ± 0.1 | -6.4 |
| CACGUG[e] | -5.8 ± 0.0 | -5.7 |
| CAGCUG[e] | -5.9 ± 0.0 | -6.7 |
| CCAUGG[e] | -6.5 ± 0.0 | -6.7 |
| CCUAGG[e] | -6.9 ± 0.0 | -6.4 |
| CUGCAG[e] | -6.2 ± 0.1 | -6.7 |
| GACGUC[e] | -6.6 ± 0.0 | -5.8 |
| GAGCUC[e] | -6.9 ± 0.1 | -6.8 |
| GCAUGC[e] | -6.9 ± 0.1 | -7.0 |

| | | |
|---|---|---|
| CGCGCG[e] | -7.7 ± 0.2 | -8.3 |
| CGGCCG[e] | -8.5 ± 0.1 | -8.9 |
| GCCGGC[e] | -10.2 ± 0.5 | -9.9 |
| GCGCGC[e] | -9.0 ± 0.4 | -9.2 |
| UAUAUAUA[e] | -2.3 ± 0.2 | -1.9 |
| UAAUAUUA[e] | -1.9 ± 0.4 | -1.2 |
| AACUAGUU[e] | -6.0 ± 0.0 | -4.5 |
| ACUAUAGU[e] | -5.8 ± 0.0 | -5.2 |
| AGAUAUCU[e] | -5.7 ± 0.1 | -5.6 |
| GAUAUAUC[e] | -5.5 ± 0.0 | -5.3 |
| GAAUAUUC[e] | -4.7 ± 0.1 | -4.6 |
| AACCGGUU[e] | -9.0 ± 0.3 | -7.6 |
| ACUGCAGU[e] | -9.2 ± 0.1 | -9.3 |
| GCAAUUGC[e] | -8.1 ± 0.1 | -8.6 |
| GAACGUUC[e] | -7.8 ± 0.1 | -7.4 |
| AGCGCGCU[e] | -12.5 ± 0.5 | -11.9 |
| AGCCGGCU[e] | -13.9 ± 0.2 | -12.5 |
| GCGAUCGC[e] | -12.4 ± 0.4 | -11.7 |
| GACCGGUC[e] | -12.9 ± 0.2 | -12.0 |
| UUAUCGAUAA[e] | -6.9 ± 0.0 | -6.8 |
| UAUCGAUA[e] | -5.8 ± 0.0 | -5.2 |
| AGCGCU[e] | -6.6 ± 0.1 | -6.4 |
| CGCGCG[e] | -8.6 ± 0.2 | -8.3 |

[a]RNA duplex consists of denoted RNA strand (5' → 3') and complementary RNA strand. [b,c]Water activities were measured to be 0.987 and 0.990 for PEG2000 and PEG8000, respectively, at their 20 wt% solution in 10 mM Na-phosphate buffer with 100 mM NaCl and 0.1 M $Na_2EDTA$. [d]20 (v/v)% PEG200 is equivalent to 21.7 (w/w)% PEG200 at 37 °C. Since the contribution of water activity and excluded volume effect will be negligibly different between 21.7 wt% and 20 wt%, we calculated $\Delta G^{\circ}_{37}$ in 20 vol% PEG200 using the parameters obtained for 20 wt% PEG200. [e]Measured values were collected from the report by Adams and Znosko.(7)

**Table S15.** Thermodynamic parameters for RNA duplexes measured in the presence of NaCl and exact intracellular cation condition with 40 wt% PEG200

| No. | Sequence[a] | 122 mM $Na^{+b}$ | | | | Intracellular cation condition[c] | | | |
|---|---|---|---|---|---|---|---|---|---|
| | | $\Delta H°$ (kcal $mol^{-1}$) | $T\Delta S°$ (cal $mol^{-1}$ $K^{-1}$) | $\Delta G°_{37}$ (kcal $mol^{-1}$) | $T_m{}^d$ (°C) | $\Delta H°$ (kcal $mol^{-1}$) | $T\Delta S°$ (cal $mol^{-1}K^{-1}$) | $\Delta G°_{37}$ (kcal $mol^{-1}$) | $T_m{}^d$ (°C) |
| NS3 | CAGCGG | -65.6 ± 3.0 | -59.2 ± 2.7 | -6.4 ± 0.4 | 36.4 | -56.5 ± 1.4 | -50.2 ± 1.3 | -6.3 ± 0.2 | 36.0 |
| NS5 | GAACUCC | -71.8 ± 2.3 | -65.9 ± 2.1 | -5.9 ± 0.3 | 34.1 | -76.9 ± 2.8 | -70.9 ± 2.6 | -6.0 ± 0.4 | 35.1 |
| NS10a | CGCUGUCG | -78.4 ± 2.4 | -68.6 ± 2.1 | -9.8 ± 1.0 | 49.8 | -74.7 ± 1.8 | -65.4 ± 1.5 | -9.3 ± 0.3 | 48.7 |
| NS11a | UGCUGUCA | -66.2 ± 1.8 | -58.4 ± 1.6 | -7.8 ± 0.7 | 43.0 | -61.0 ± 2.3 | -53.6 ± 2.0 | -7.4 ± 0.4 | 41.6 |
| NS13a | GUGCCGAG | -81.3 ± 1.3 | -70.5 ± 1.1 | -10.8 ± 0.5 | 54.3 | -73.1 ± 4.6 | -62.9 ± 4.0 | -10.2 ± 0.8 | 53.0 |
| NS13b | GCCGAGUG | -71.6 ± 2.1 | -61.2 ± 1.8 | -10.4 ± 0.8 | 54.1 | -74.1 ± 3.2 | -63.8 ± 2.7 | -10.3 ± 0.5 | 53.6 |
| S2 | GGUAUACC | -71.9 ± 3.9 | -65.7 ± 3.6 | -6.2 ± 0.5 | 39.2 | -73.4 ± 1.4 | -67.5 ± 1.3 | -5.9 ± 0.2 | 37.9 |
| S3 | GGUUAACC | -73.4 ± 2.8 | -67.7 ± 2.6 | -5.7 ± 0.4 | 36.5 | -68.5 ± 2.9 | -63.1 ± 2.5 | -5.4 ± 0.4 | 35.4 |
| S5 | GCUUAAGC | -70.4 ± 3.2 | -64.3 ± 2.9 | -6.1 ± 0.4 | 38.6 | -74.0 ± 5.1 | -68.7 ± 4.8 | -5.3 ± 0.7 | 35.4 |
| S7 | CGCAUGCG | -79.3 ± 3.7 | -69.7 ± 3.2 | -9.6 ± 0.6 | 52.7 | -78.1 ± 5.2 | -69.3 ± 4.6 | -8.8 ± 0.9 | 50.3 |
| S8 | CGGUACCG | -73.4 ± 6.2 | -64.4 ± 5.5 | -9.0 ± 1.0 | 52.7 | -75.0 ± 1.7 | -65.9 ± 1.5 | -9.1 ± 0.3 | 52.0 |
| S11a | GAUCCGGAUC | -103.9 ± 1.7 | -91.6 ± 1.5 | -12.3 ± 0.7 | 58.4 | -94.8 ± 3.3 | -83.3 ± 2.9 | -11.5 ± 0.5 | 57.0 |
| S12a | AUGAGCUCAU | -80.7 ± 2.3 | -72.1 ± 2.0 | -8.6 ± 0.3 | 48.8 | -80.9 ± 5.8 | -72.8 ± 5.2 | -8.1 ± 0.7 | 46.8 |
| S12b | AUCAGCUGAU | -83.3 ± 1.3 | -75.1 ± 1.1 | -8.2 ± 0.5 | 46.7 | -81.9 ± 1.9 | -74.2 ± 1.8 | -7.7 ± 0.3 | 44.7 |
| S13 | UUACGCGUAA | -78.9 ± 4.7 | -71.7 ± 4.2 | -7.2 ± 0.6 | 42.2 | -75.7 ± 2.2 | -68.8 ± 2.0 | -6.9 ± 0.3 | 41.7 |
| S14 | AUCGCUAGCGAU | -105.3 ± 4.7 | -92.3 ± 4.8 | -13.0 ± 0.8 | 60.0 | -95.0 ± 8.3 | -83.4 ± 7.3 | -11.6 ± 1.5 | 57.1 |

[a]RNA duplex consists of denoted RNA strand (5′ → 3′) and complementary RNA strand. [b]Experiments were performed in a buffer containing 100 mM NaCl, 10 mM $Na_2HPO_4$, and 1 mM $Na_2EDTA$ in 40 wt% PEG200 at pH 7.0. Values were collected from Table S2. [c]Experiments were performed in a buffer containing 120 mM KCl, 10 mM NaCl, 0.5 mM $MgCl_2$, 0.0001 mM $CaCl_2$ and 10 mM $K_2HPO_4$ in 40 wt% PEG200 at pH 7.2. [d]Melting temperatures were calculated for total strand concentration of 100 µM.

**Table S16.** Measured thermodynamic parameters for RNA duplexes in exact intracellular cation condition and the predicted values using parameters in 40 wt% PEG200 with 100 mM NaCl

| | | Measured[b] | | | | Predicted[c] | | | |
|---|---|---|---|---|---|---|---|---|---|
| No. | Sequence[a] | $\Delta H°$ (kcal mol$^{-1}$) | $\Delta S°$ (cal mol$^{-1}$ K$^{-1}$) | $\Delta G°_{37}$ (kcal mol$^{-1}$) | $T_m$[d] (°C) | $\Delta H°$ (kcal mol$^{-1}$) | $\Delta S°$ (cal mol$^{-1}$K$^{-1}$) | $\Delta G°_{37}$ (kcal mol$^{-1}$) | $T_m$[d] (°C) |
| NS3 | CAGCGG | -56.5 | -161.9 | -6.3 | 36.0 | -58.4 | -167.2 | -6.5 | 37.1 |
| NS5 | GAACUCC | -76.9 | -228.6 | -6.0 | 35.1 | -65.5 | -191.8 | -6.0 | 34.6 |
| NS10a | CGCUGUCG | -74.7 | -210.9 | -9.3 | 48.7 | -77.1 | -216.8 | -9.8 | 51.0 |
| NS11a | UGCUGUCA | -61.0 | -172.8 | -7.4 | 41.6 | -66.1 | -187.0 | -8.1 | 44.6 |
| NS13a | GUGCCGAG | -73.1 | -202.8 | -10.2 | 53.0 | -80.8 | -226.4 | -10.6 | 53.4 |
| NS13b | GCCGAGUG | -74.1 | -205.7 | -10.3 | 53.6 | -80.8 | -226.4 | -10.6 | 53.4 |
| S2 | GGUAUACC | -73.4 | -217.6 | -5.9 | 37.9 | -78.7 | -232.3 | -6.7 | 40.9 |
| S3 | GGUUAACC | -68.5 | -203.4 | -5.4 | 35.4 | -77.5 | -230.8 | -5.9 | 38.0 |
| S5 | GCUUAAGC | -74.0 | -221.5 | -5.3 | 35.4 | -76.5 | -226.6 | -6.2 | 39.2 |
| S7 | CGCAUGCG | -78.1 | -233.4 | -8.8 | 50.3 | -79.5 | -226.1 | -9.4 | 52.1 |
| S8 | CGGUACCG | -75.0 | -212.5 | -9.1 | 52.0 | -79.7 | -227.6 | -9.1 | 51.0 |
| S11a | GAUCCGGAUC | -94.8 | -268.6 | -11.5 | 57.0 | -103.1 | -293.5 | -12.1 | 57.5 |
| S12a | AUGAGCUCAU | -80.9 | -234.7 | -8.1 | 46.8 | -86.4 | -250.1 | -8.8 | 48.8 |
| S12b | AUCAGCUGAU | -81.9 | -239.2 | -7.7 | 44.7 | -86.4 | -250.1 | -8.8 | 48.8 |
| S13 | UUACGCGUAA | -75.7 | -221.8 | -6.9 | 41.7 | -82.0 | -241.3 | -7.2 | 42.7 |
| S14 | AUCGCUAGCGAU | -95.0 | -268.9 | -11.6 | 57.1 | -109.3 | -309.6 | -13.3 | 60.2 |

[a]RNA duplex consists of denoted RNA strand (5′ → 3′) and complementary RNA strand. [b]Errors associated with the measured values were mentioned in the Table S15. [c]Predicted using the parameters of Table 1. [d]Melting temperatures were calculated for total strand concentration of 100 μM.

**Table S17.** Parameters for $\Delta G°_{\text{NN, [no cosolute, 122 mM Na}^+]}$, $\Delta G°_{\text{NN, ev}}$, $\Delta G°_{\text{NN, wa}}$ and prefactor of PEG ($m_{\text{PEG}}$) at 25 °C

| Sequence | $\Delta G°_{\text{25, NN, [no cosolute, 122 mM Na}^+]}$[a] (kcal mol$^{-1}$) | $\Delta G°_{\text{25, NN, ev [40 wt\% PEG200]}}$[b] (kcal mol$^{-1}$) | $\Delta G°_{\text{25, NN, wa [40 wt\% PEG200]}}$[c] (kcal mol$^{-1}$) | $m_{\text{PEG}}$[d] (kcal mol$^{-1}$) |
|---|---|---|---|---|
| r(AA/UU) | -1.10 | -0.21 | 0.37 | 7.6 |
| r(AU/UA) | -0.81 | -0.21 | 0.09 | 1.9 |
| r(UA/AU) | -1.70 | -0.21 | 0.19 | 3.9 |
| r(CA/GU) | -2.16 | -0.21 | -0.16 | -3.3 |
| r(GU/CA) | -2.50 | -0.21 | 0.56 | 11.4 |
| r(CU/GA) | -2.12 | -0.21 | 0.19 | 3.9 |
| r(GA/CU) | -2.70 | -0.21 | 0.30 | 6.1 |
| r(CG/GC) | -2.53 | -0.21 | 0.22 | 4.5 |
| r(GC/CG) | -3.78 | -0.21 | 0.50 | 10.1 |
| r(GG/CC) | -3.48 | -0.21 | 0.34 | 6.9 |
| initiation | 4.05 | -0.21 | 1.63 | 33.2 |
| per terminal AU | 0.57 | NA | 0.51 | 10.4 |

[a]Calculated from the $\Delta H°$ and $\Delta S°$ values in Table S3. [b]$\Delta G°_{\text{25, NN, ev [40 wt\% PEG200]}}$ were calculated similarly as done in Table 2. [c]$\Delta G°_{\text{25, NN, wa [40 wt\% PEG200]}} = \Delta G°_{\text{25, NN, [40 wt\% PEG200, 122 mM Na}^+]} - \Delta G°_{\text{25, NN, [no cosolute, 122 mM Na}^+]} - \Delta G°_{\text{25, NN, ev [40 wt\% PEG200]}}$. $\Delta G°_{\text{25, NN, [40 wt\% PEG200, 122 mM Na}^+]}$ were calculated from the $\Delta H°$ and $\Delta S°$ values in Table 1. [d]Determined by using equation 13.

**Table S18**. Calculated $\Delta G°_{25,\ [crowder]}$ for $(ACUG)_3$ and $(ACUG)_6$ in various PEG200 concentrations at 25 °C in 100 mM NaCl

| Solution | $(ACUG)_3$ | | | $(ACUG)_6$ | | |
|---|---|---|---|---|---|---|
| | $\Delta G°_{25,\ wa}$[a] (kcal mol$^{-1}$) | $\Delta G°_{25,\ ev}$[b] (kcal mol$^{-1}$) | $\Delta G°_{25,\ [crowder]}$[c] (kcal mol$^{-1}$) | $\Delta G°_{25,\ wa}$[a] (kcal mol$^{-1}$) | $\Delta G°_{25,\ ev}$[b] (kcal mol$^{-1}$) | $\Delta G°_{25,\ [crowder]}$[c] (kcal mol$^{-1}$) |
| 10 wt% PEG200 | 1.0 | -0.6 | 0.4 | 1.6 | -1.0 | 0.6 |
| 20 wt% PEG200 | 2.3 | -1.2 | 1.1 | 3.7 | -2.0 | 1.7 |
| 30 wt% PEG200 | 3.7 | -1.7 | 2.0 | 5.8 | -3.0 | 2.8 |
| 40 wt% PEG200 | 4.5 | -2.3 | 2.2 | 7.2 | -4.0 | 3.2 |
| 50 wt% PEG200 | 6.0 | -2.9 | 3.1 | 9.5 | -5.1 | 4.4 |

[a]$\Delta G°_{25,\ wa}$ were calculated from the $m_{PEG}$ values in Table S17 and $\Delta a_w$ values using equation 13 at 298 K. [b]$\Delta G°_{25,\ ev}$ were calculated using equation 11 at 298 K.[c]$\Delta G°_{25,[crowder]} = \Delta G°_{25,\ wa} + \Delta G°_{25,\ ev.}$.

**Table S19**. Estimated NN parameters including G-U wobble base pairs present in Im-4U

| Sequence[a] | $\Delta G^\circ_{37\ \mathrm{NN,\ [no,\ cosolute,\ 122\ mM\ Na^+]}}$ (kcal mol$^{-1}$) | $m_{\mathrm{PEG/2\text{-}ME/1,2\ DME}}$ (kcal mol$^{-1}$) | $m_{\mathrm{EG/Gly/1,3\ PDO}}$ (kcal mol$^{-1}$) |
|---|---|---|---|
| r(GA/UU) | -0.96 | 7.1 | 2.9 |
| r(GG/UU) | -0.38 | 7.1 | 2.9 |
| r(AG/UU) | -0.41 | 7.1 | 2.9 |
| r(AU/UG) | -0.64 | 3.9 | 1.6 |
| r(UG/AU) | -0.98 | 3.9 | 1.6 |

[a]$\Delta G^\circ_{37}$ in 1 M NaCl solution were collected from collected from the report by Turner et al. (1)

**Table S20.** $\Delta G°_{37}$ of GAAA tetraloop in the absence and presence of 40 wt% PEG200

| Sequence[a] | In the absence of PEG200[b] (kcal $mol^{-1}$) | In the presence of 40 wt% PEG200[b] (kcal $mol^{-1}$) |
|---|---|---|
| 5′GGAG<u>GAAA</u>CUCC 3′ | 1.47 ± 0.06 | 0.63 ± 0.09 |
| 5′GAAG<u>GAAA</u>CUUC 3′ | 1.36 ± 0.06 | 0.57 ± 0.09 |

[a]Underlined region is the loop sequence. [b]Values were collected from the report by Leonard and Blose.(3)

**Table S21.** Predicted $\Delta G°_{37}$ of RNA hairpin Im-4U in different cosolute concentrations at 100 mM NaCl along with the $\Delta a_w$ values in those cosolute solutions

| Solution | $\Delta a_w$ | $\Delta G°_{37}$[a] (kcal mol$^{-1}$) |
|---|---|---|
| 10 wt% EG | 0.026 | -0.5 |
| 20 wt% EG | 0.047 | -1.3 |
| 30 wt% EG | 0.072 | -2.2 |
| 40 wt% EG | 0.087 | -2.8 |
| 10 wt% Gly | 0.021 | -0.2 |
| 20 wt% Gly | 0.045 | -1.0 |
| 30 wt% Gly | 0.076 | -2.0 |
| 40 wt% Gly | 0.101 | -2.8 |
| 10 wt% 1,3 PDO | 0.025 | -0.4 |
| 20 wt% 1,3 PDO | 0.049 | -1.2 |
| 30 wt% 1,3 PDO | 0.066 | -1.9 |
| 40 wt% 1,3 PDO | 0.089 | -2.7 |

[a]$\Delta G°_{37} = \Delta G°_{37,\text{ no cosolute, 100 mM NaCl}} + \Delta G°_{37,\text{ wa}} + \Delta G°_{37,\text{ ev}}$, as shown in Figure S6. $\Delta G°_{37,\text{ no cosolute, 100 mM NaCl}}$ were calculated from the data in Table 2 and Table S19. $\Delta G°_{37,\text{ wa}}$ were determined from the $m_{cs}$ values in Table S19 and $\Delta a_w$ values in this Table and equation 13. $\Delta G°_{37,\text{ loop, [1M NaCl]}}$ values were collected from the report of Turner et al.(1) $\Delta G°_{37,\text{ ev}}$ was determined from the data in Table S6 and equation 8. For hairpin, $\Delta V = V_{\text{hairpin}} - V_{\text{single strand}}$.

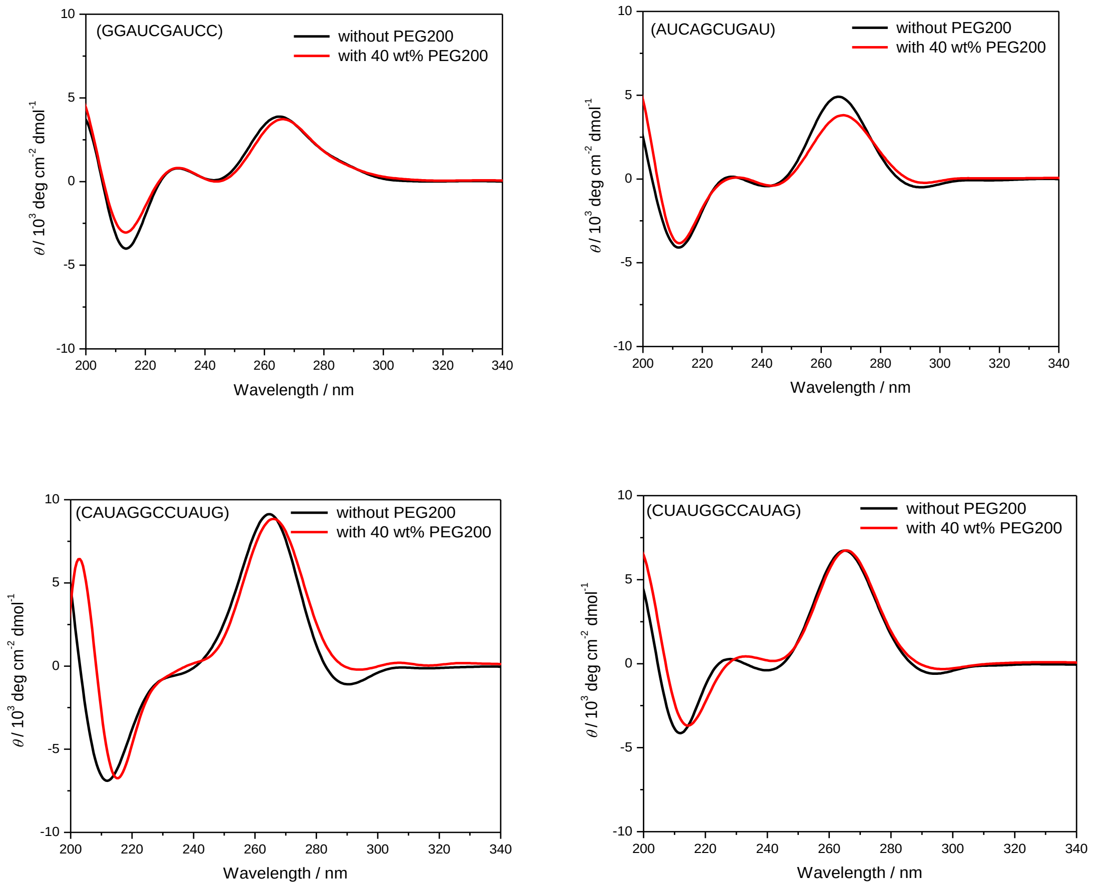


**Figure S1**. Representative CD spectra of 20 μM RNA oligonucleotides at 4 °C in a buffer containing 0.1 M NaCl, 10 mM $Na_2HPO_4$ (pH 7.0) and 1 mM $Na_2EDTA$ in the absence (black) and presence (red) of 40 wt% PEG200.

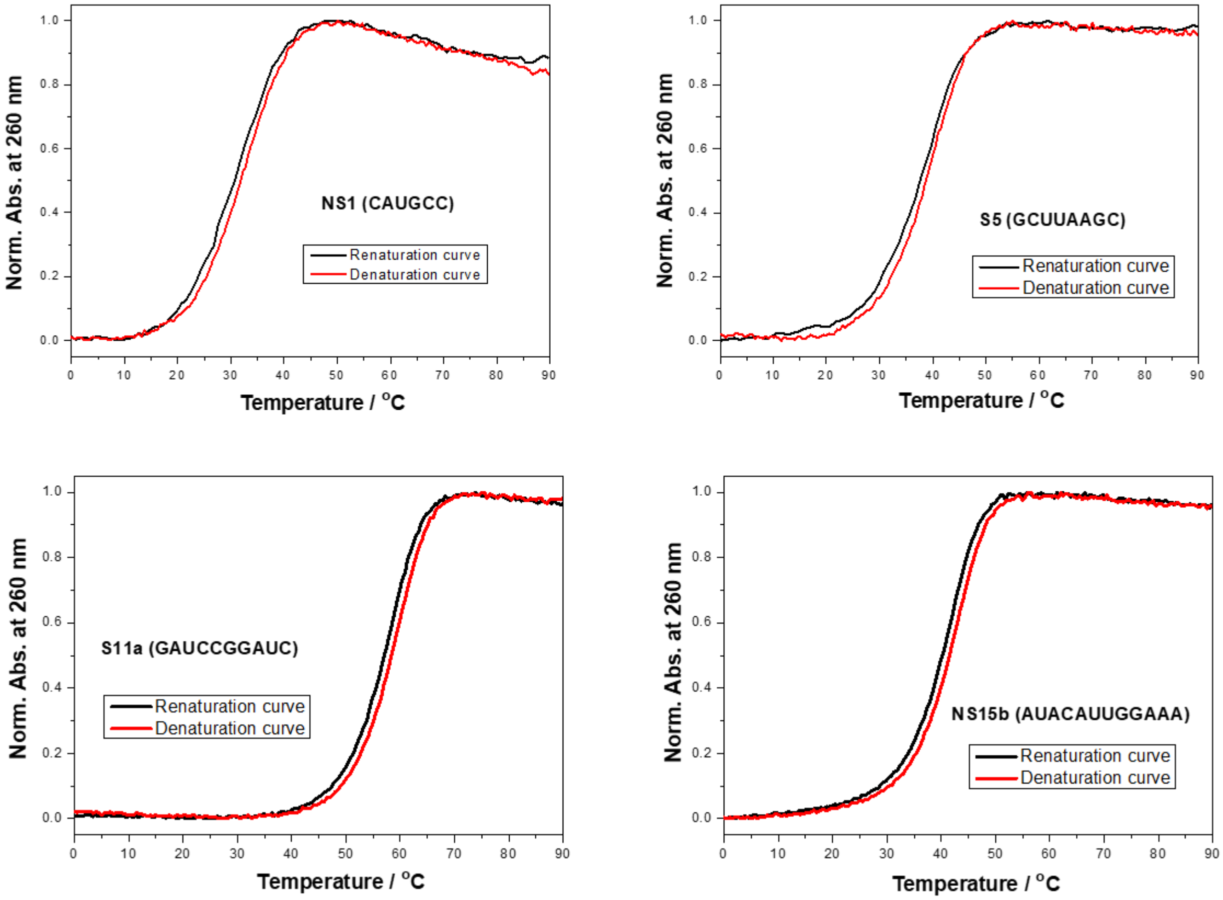


**Figure S2**. Renaturation and denaturation curves for some representative RNA oligonucleotides at 100 μM concentration in a buffer containing 0.1 M NaCl, 10 mM $Na_2HPO_4$ (pH 7.0), 1 mM $Na_2EDTA$ and 40 wt% PEG200.

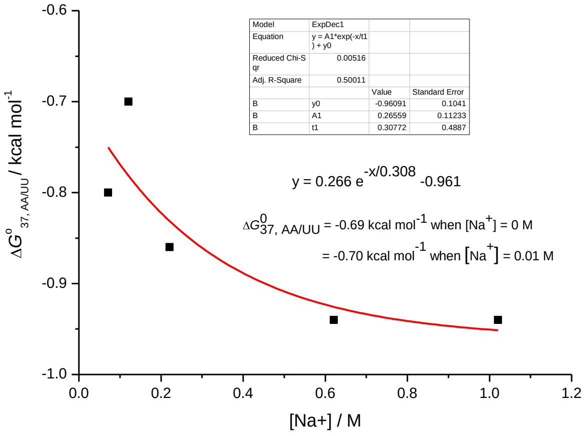
Model ExpDec1
Equation y = A1*exp(-x/t1) + y0
Reduced Chi-Sqr 0.00516
Adj. R-Square 0.50011
Value Standard Error
B y0 -0.96091 0.1041
B A1 0.26559 0.11233
B t1 0.30772 0.4887
y = 0.266 e^-x/0.308 -0.961
ΔG037, AA/UU = -0.69 kcal mol-1 when [Na+] = 0 M
= -0.70 kcal mol-1 when [Na+] = 0.01 M
ΔGo37, AA/UU / kcal mol-1
[Na+] / M

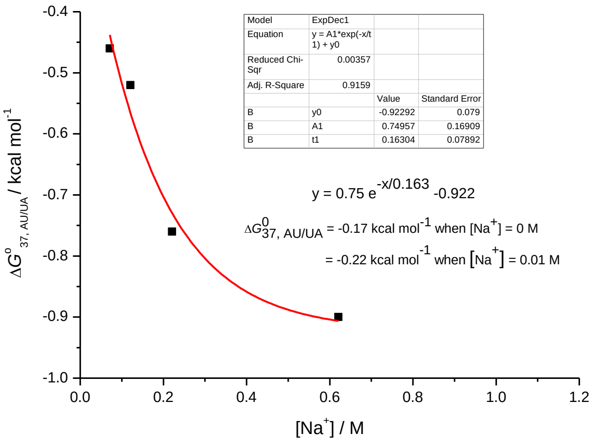
Model ExpDec1
Equation y = A1*exp(-x/t1) + y0
Reduced Chi-Sqr 0.00357
Adj. R-Square 0.9159
Value Standard Error
B y0 -0.92292 0.079
B A1 0.74957 0.16909
B t1 0.16304 0.07892
y = 0.75 e^-x/0.163 -0.922
ΔG037, AU/UA = -0.17 kcal mol-1 when [Na+] = 0 M
= -0.22 kcal mol-1 when [Na+] = 0.01 M
ΔGo37, AU/UA / kcal mol-1
[Na+] / M

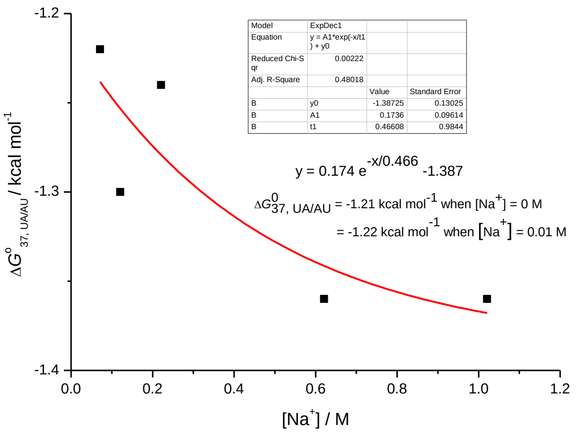
Model ExpDec1
Equation y = A1*exp(-x/t1) + y0
Reduced Chi-Sqr 0.00222
Adj. R-Square 0.48018
Value Standard Error
B y0 -1.38725 0.13025
B A1 0.1736 0.09614
B t1 0.46608 0.9844
y = 0.174 e^-x/0.466 -1.387
ΔG037, UA/AU = -1.21 kcal mol-1 when [Na+] = 0 M
= -1.22 kcal mol-1 when [Na+] = 0.01 M
ΔGo37, UA/AU / kcal mol-1
[Na+] / M

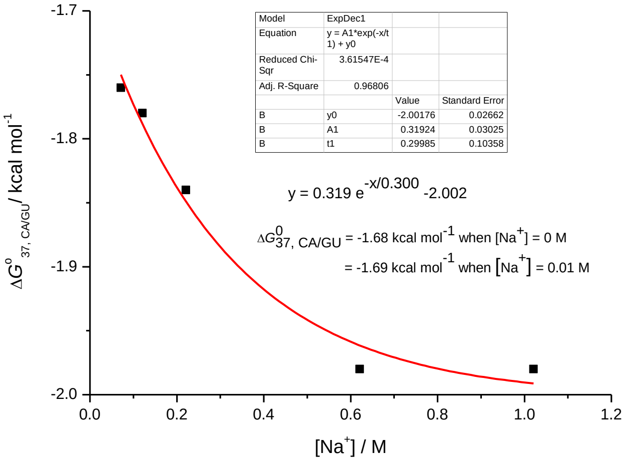
Model ExpDec1
Equation y = A1*exp(-x/t1) + y0
Reduced Chi-Sqr 3.61547E-4
Adj. R-Square 0.96806
Value Standard Error
B y0 -2.00176 0.02662
B A1 0.31924 0.03025
B t1 0.29985 0.10358
y = 0.319 e^-x/0.300 -2.002
ΔG037, CA/GU = -1.68 kcal mol-1 when [Na+] = 0 M
= -1.69 kcal mol-1 when [Na+] = 0.01 M
ΔGo37, CA/GU / kcal mol-1
[Na+] / M

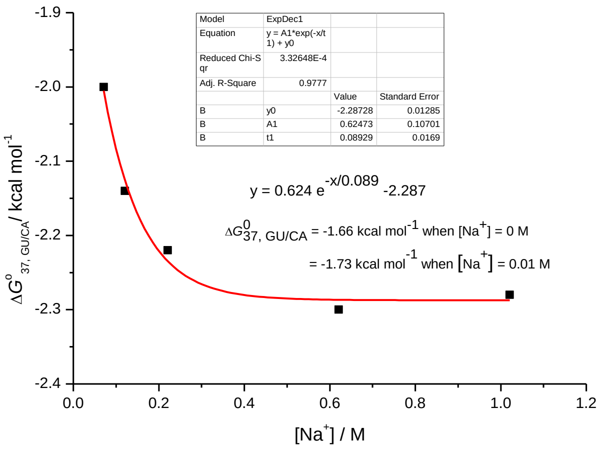
Model ExpDec1
Equation y = A1*exp(-x/t1) + y0
Reduced Chi-Sqr 3.32648E-4
Adj. R-Square 0.9777
Value Standard Error
B y0 -2.28728 0.01285
B A1 0.62473 0.10701
B t1 0.08929 0.0169
y = 0.624 e^-x/0.089 -2.287
ΔG037, GU/CA = -1.66 kcal mol-1 when [Na+] = 0 M
= -1.73 kcal mol-1 when [Na+] = 0.01 M
ΔGo37, GU/CA / kcal mol-1
[Na+] / M

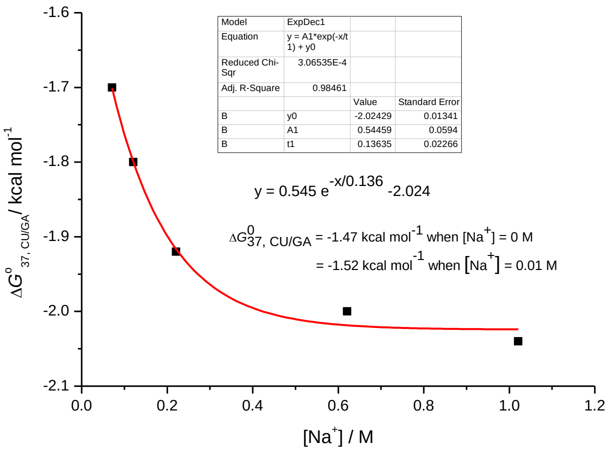
Model ExpDec1
Equation y = A1*exp(-x/t1) + y0
Reduced Chi-Sqr 3.06535E-4
Adj. R-Square 0.98461
Value Standard Error
B y0 -2.02429 0.01341
B A1 0.54459 0.0594
B t1 0.13635 0.02266
y = 0.545 e^-x/0.136 -2.024
ΔG037, CU/GA = -1.47 kcal mol-1 when [Na+] = 0 M
= -1.52 kcal mol-1 when [Na+] = 0.01 M
ΔGo37, CU/GA / kcal mol-1
[Na+] / M

Continued

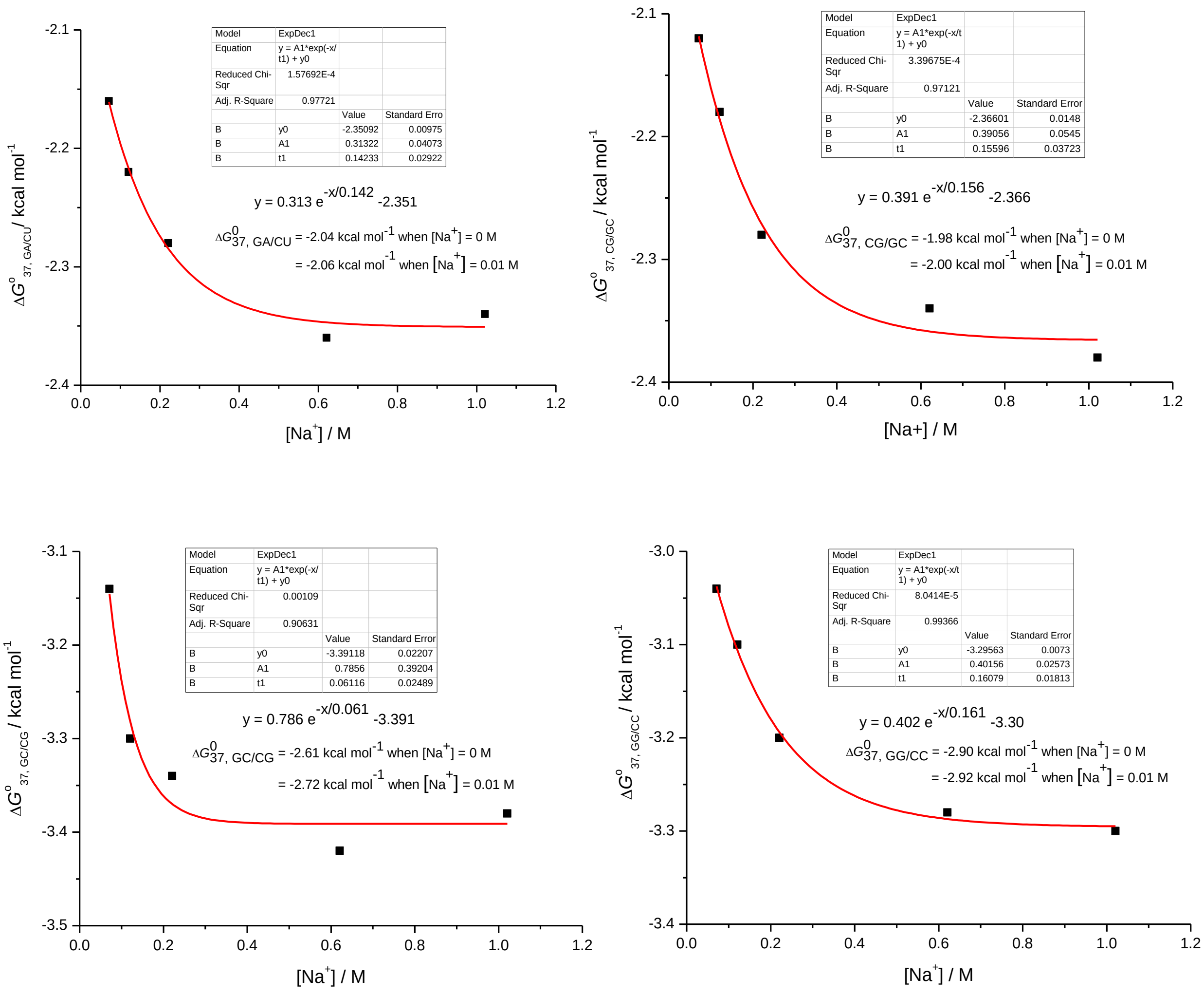


**Figure S3**. Plot of individual $\Delta G^{\circ}_{37,\,\mathrm{NN}}$ against the concentration of $Na^{+}$. Values are collected from the report by Weber et al.(4)

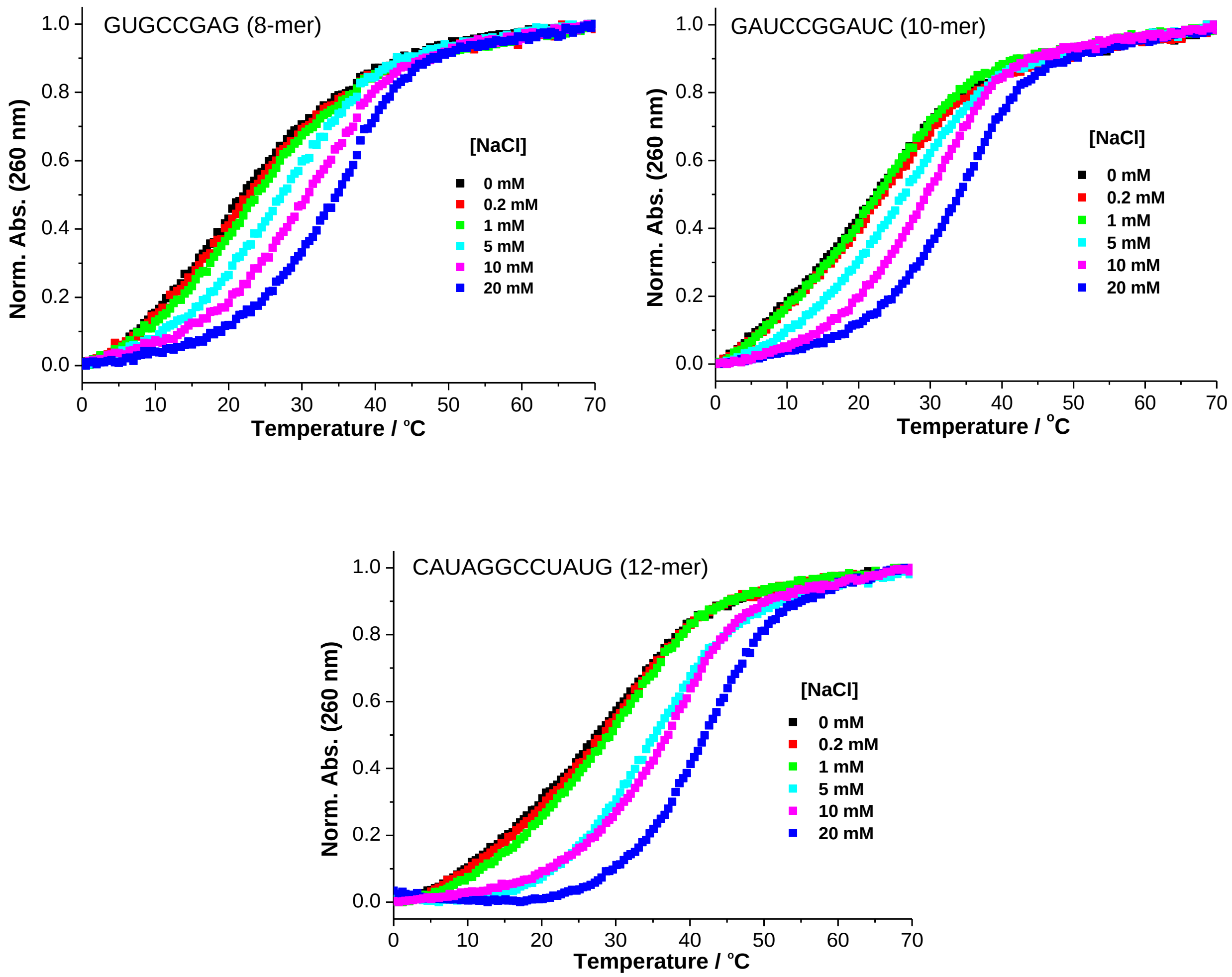


**Figure S4**. UV melting profiles of 10 µM RNA duplexes with the varying concentrations of NaCl. Samples were dialyzed overnight in Milli-Q water before setting the cation concentrations.

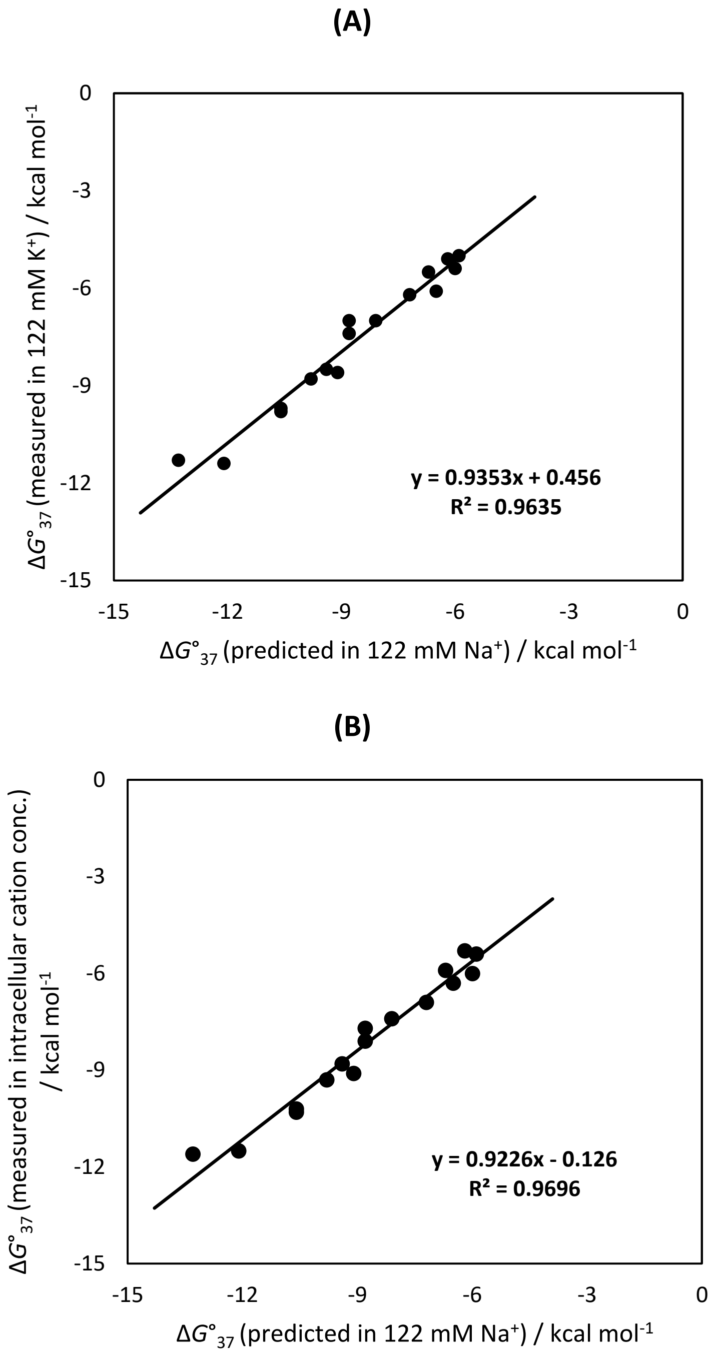


**Figure S5**. Relationship between measured $\Delta G°_{37}$ of RNA duplexes in **(A)** 122 mM $K^+$ solution or **(B)** intracellular cation concentration and predicted $\Delta G°_{37}$ in 122 mM $Na^+$ solution under the crowding condition of 40 wt% PEG200. Measured values are provided in Table S9 and Table S15, respectively and predicted values collected from Table S5.

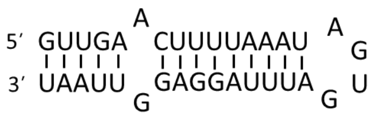


Im-4U

$$\Delta G^{\circ}_{37,\text{ hairpin}} = 3\Delta G^{\circ}_{37}\,(\text{AA/UU}) + \Delta G^{\circ}_{37}\,(\text{CU/GA}) + \Delta G^{\circ}_{37}\,(\text{AU/UA}) + \Delta G^{\circ}_{37}\,(\text{UA/AU}) + 2\Delta G^{\circ}_{37}\,(\text{GA/UU}) + \Delta G^{\circ}_{37}\,(\text{GG/UU}) + \Delta G^{\circ}_{37}\,(\text{AG/UU}) + \Delta G^{\circ}_{37}\,(\text{AU/UG}) + \Delta G^{\circ}_{37}\,(\text{UG/AU}) + \Delta G^{\circ}_{37,\text{ initiation}} + \Delta G^{\circ}_{37,\text{ terminal GU}} + \Delta G^{\circ}_{37,\text{ hairpin loop}} + \Delta G^{\circ}_{37,\text{ internal loop}}$$

$$\Delta G^{\circ}_{37}\,(\text{pred.}) = \Delta G^{\circ}_{37,\text{ hairpin, no cosolute}} + \Delta G^{\circ}_{37,\text{ hairpin, wa}} + \Delta G^{\circ}_{37,\text{ hairpin, ev}}$$

$\Delta G^{\circ}_{37}\,(\text{AA/UU}) = \{ -0.70 + (7.1 \times 0.049)\}$ $\Delta G^{\circ}_{37}\,(\text{CU/GA}) = \{ -1.80 + (5.1 \times 0.049)\}$

$\Delta G^{\circ}_{37}\,(\text{AU/UA}) = \{ -0.52 + (3.9 \times 0.049)\}$ $\Delta G^{\circ}_{37}\,(\text{UA/AU}) = \{ -1.30 + (3.9 \times 0.049)\}$

$\Delta G^{\circ}_{37}\,(\text{GA/UU}) = \{ -0.96 + (7.1 \times 0.049)\}$ $\Delta G^{\circ}_{37}\,(\text{GG/UU}) = \{ -0.38 + (7.1 \times 0.049)\}$

$\Delta G^{\circ}_{37}\,(\text{AG/UU}) = \{ -0.41 + (7.1 \times 0.049)\}$ $\Delta G^{\circ}_{37}\,(\text{AU/UG}) = \{ -0.64 + (3.9 \times 0.049)\}$

$\Delta G^{\circ}_{37}\,(\text{UG/AU}) = \{ -0.98 + (3.9 \times 0.049)\}$ $\Delta G^{\circ}_{37,\text{ initiation}} = \{ 4.09 + (33.3 \times 0.049)\}$

$\Delta G^{\circ}_{37,\text{ terminal GU}} = \{ 0.45 + (8.2 \times 0.049)\}$ $\Delta G^{\circ}_{37,\text{ hairpin loop}} = \{4.95 - (11.8 \times 5.25 \times 0.049)\}$

$\Delta G^{\circ}_{37,\text{ internal loop}} = \{1.05 - (11.8 \times 1.05 \times 0.049)\}$ $\Delta G^{\circ}_{37,\text{ hairpin, ev}} = -1.42$

$\Delta G^{\circ}_{37}\,(\text{pred.}) = 1.1\ \text{kcal mol}^{-1}$

$\Delta G^{\circ}_{37}\,(\text{exp.}) = 0.4\ \text{kcal mol}^{-1}$

**Figure S6.** Secondary structure of RNA hairpin Im-4U and prediction of its stability in 40 wt% PEG200 at 100 mM NaCl.

**References**


1. Mathews, D.H., Sabina, J., Zuker, M. and Turner, D.H. (1999) Expanded sequence dependence of thermodynamic parameters improves prediction of RNA secondary structure11Edited by I. Tinoco. *J. Mol. Biol.*, **288**, 911-940.
2. Tan, Z.-J. and Chen, S.-J. (2008) Salt Dependence of Nucleic Acid Hairpin Stability. *Biophys. J.*, **95**, 738-752.
3. Leonard, K.N. and Blose, J.M. (2018) Effects of osmolytes and macromolecular crowders on stable GAAA tetraloops and their preference for a CG closing base pair. *PeerJ*, **6**, e4236-e4236.
4. Ferreira, I., Jolley, E.A., Znosko, B.M. and Weber, G. (2019) Replacing salt correction factors with optimized RNA nearest-neighbour enthalpy and entropy parameters. *Chem. Phys.*, **521**, 69-76.
5. Knowles, D.B., LaCroix, A.S., Deines, N.F., Shkel, I. and Record, M.T. (2011) Separation of preferential interaction and excluded volume effects on DNA duplex and hairpin stability. *Proc. Natl. Acad. Sci. U. S. A.*, **108**, 12699-12704.
6. Liu, J.-H., Xi, K., Zhang, X., Bao, L., Zhang, X. and Tan, Z.-J. (2019) Structural Flexibility of DNA-RNA Hybrid Duplex: Stretching and Twist-Stretch Coupling. *Biophys. J.*, **117**, 74-86.
7. Adams, M.S. and Znosko, B.M. (2019) Thermodynamic characterization and nearest neighbor parameters for RNA duplexes under molecular crowding conditions. *Nucleic Acids Res.*, **47**, 3658-3666.
